# Insights into *Mycobacterium abscessus* survival under prolonged potassium deficiency and starvation

**DOI:** 10.3389/fcimb.2025.1668407

**Published:** 2025-11-26

**Authors:** Artem S. Grigorov, Billy A. Martini, Vladimir V. Sorokin, Tatyana L. Azhikina, Andrey L. Mulyukin, Elena G. Salina

**Affiliations:** 1 Shemyakin and Ovchinnikov Institute of Bioorganic Chemistry, Russian Academy of Sciences, Moscow, Russia; 2 Bach Institute of Biochemistry, Research Center of Biotechnology of the Russian Academy of Sciences, Moscow, Russia; 3 Winogradsky Institute of Microbiology, Research Center of Biotechnology of the Russian Academy of Sciences, Moscow, Russia

**Keywords:** *Mycobacterium abscessus*, non-tuberculous mycobacteria, dormancy, starvation, non-replicative state, survival, cation homeostasis

## Abstract

*Mycobacterium abscessus* (Mab) is known for its ability to cause chronic infections, to be resistant to antimicrobial agents and to survive for extended periods in different non-replicative states (NRS), including persistence, dormancy or starvation. Functional metabolic pathways for Mab surviving in particular NRS caused by potassium depletion or by starvation, which are the conditions common in infected hosts or natural environments, remains unexplored. Dormant and starved Mab cultures were able to maintain viability, exhibiting decreased ^3^H‐uracil incorporation and altered cell ultrastructure compared to actively growing cells. Specifically, dormant Mab populations were heterogeneous in the ability to cope with potassium deficiency, either maintaining very low or near-normal K^+^-levels, or capturing other cations. Transcriptome and proteome profiling revealed both common and specific metabolic reprogramming in dormant and starved Mab, including downregulation of the major biosynthetic pathways and upregulation of β-oxidation of fatty acid. Specifically, dormant Mab cells were enriched in the dormancy regulator DosR and the potassium-transporting Kdp system, corresponding to their enhanced transcription. Unlike dormant Mab, starved Mab contained an elevated pool of proteins underrepresented in transcriptome, such as the DNA-binding histone-like protein and the universal stress proteins. In dormant Mab, up- or down-regulation at the transcriptional and translational level matches better than in starved cells. Notably, transcripts and proteins of the MmpL and MmpS family, which are associated with mycobacterial virulence, and lipid-transporting Mce proteins, which modulate host-cell signaling, were depleted in the both dormant and starved Mab. Overall, the results of this study provide insight into molecular mechanisms by which Mab adapts to clinically relevant and long-term environmental stresses and survives in NRS.

## 1 Introduction

*Mycobacterium abscessus* (Mab) is a fast-growing, non-tuberculous mycobacterium that is notorious for its resistance to antimicrobial agents and its ability to cause chronic infections (Johansen et al., 2020; Cocorullo et al., 2025). Like Koch’s bacillus, Mab can cause latent infections and persist in the host for extended periods, sometimes for decades, before causing acute infections (Medjahed et al., 2010; Viljoen et al., 2016) and are capable of surviving in human macrophages (Roux et al., 2016; Viljoen et al., 2016; Dubois et al., 2019; Johansen et al., 2020; Touré et al., 2023). Latent mycobacterial infections have been associated with dormancy (Zhang, 2004; Lewis, 2007; Chao and Rubin, 2010; Verma et al., 2022), a state of reduced metabolic activity and growth arrest, enabling bacteria to survive in different niches. The mechanisms by which Mab persists in the host organism are not understood; only the recent study has suggested that this bacterium adopts a dormant-like phenotype within lipid droplets of adipocytes (Kurihara et al., 2025).

When Mab enters a non-replicative state (NRS), it becomes much less susceptible or absolutely tolerant to many clinically used antibiotics, as demonstrated in comprehensive *in vitro* assays with several NRS types and biofilms (Yam et al., 2020), or with disparate models, including starvation in phosphate buffer (Berube et al., 2018), hypoxia (Feilcke et al., 2025), and prolonged incubation in potassium-free medium (Mulyukin et al., 2023). The development or adoption of NRS models is currently needed to find agents that will be effective in combatting persistent Mab and treating latent infections caused by this mycobacterium (Yam et al., 2020). Furthermore, the above-mentioned studies have already demonstrated *in vitro* the effect of some new antimicrobials against non-replicating Mab.

The molecular mechanisms underlying Mab survival and adaptation to a number of clinically relevant conditions or stressful factors have been explored not as comprehensively as those of *M. tuberculosis* (Mtb) non-replicative state (Betts et al., 2002; Bacon et al., 2004; Voskuil et al., 2004; Taneja et al., 2010; Salina et al., 2014a; Ignatov et al., 2015; Aguilar-Ayala et al., 2017). Transcriptional responses of Mab were examined upon exposure to NO-induced hypoxia, erythromycin or kanamycin, or artificial cystic fibrosis (CF) sputum (Miranda-CasoLuengo et al., 2016); hypoxia (Simcox et al., 2023); some transition metal cations (Co^2+^ and Ni^2+^) (Liu et al., 2024) and upon intracellular survival in amoebae or murine macrophages (Dubois et al., 2019).

In this study, we focused on two NRS models: the aged post-stationary Mab cultures grown under potassium depletion and the starved cell suspensions in phosphate buffer. Both potassium deficiency (Tan, 2021) and starvation (Berube et al., 2018) can occur, on the one hand, in the local milieu of the host. On the other hand, these conditions are common for natural ecosystems, from which several Mab strains have been isolated and characterized (Vang et al., 2022). Moreover, potassium deficiency can be observed in the phagosome due to the operation of a K^+^-efflux pump (Wiese and Seydel, 1996; Amaral et al., 2007). Potassium is known to be capable of controlling host colonization in many bacterial species including Mtb (MacGilvary et al., 2019; Liu et al., 2013; Gries et al., 2013; Stingl et al., 2007).

However, dormant mycobacteria are particular models for omics studies, since the acquirement and maturation of a NRS typically require specific conditions and a prolonged period of time, as it was demonstrated for Mtb (Hampshire et al., 2004; Taneja et al., 2010; Shleeva et al., 2011; Salina et al., 2014a; Ignatov et al., 2015) and *M. smegmatis* (Anuchin et al., 2009; Mulyukin et al., 2010; Shleeva et al., 2017; Trutneva et al., 2018). Phenotypically, the Mab’s response to the prolonged incubation under K^+^-deficiency involved entering a particular state of dormancy in which the major population retained the ability to reactivate in a fresh nutritionally complete liquid medium but not on nutrient agar (Mulyukin et al., 2023). To the best of our knowledge, the functional networks and active or conserved metabolic pathways in dormant and starved Mab remain unexplored. Based on our studies with Mtb (Ignatov et al., 2015), it can be assumed that *de novo* mRNA synthesis may be significantly attenuated in non-replicating Mab with substantial decrease in the number of transcripts in comparison to actively growing bacteria. We hypothesize that dormant and starved Mab will possess either common or specific sets of differentially regulated genes and proteins, similar to what has already been demonstrated for various dormant Mtb models (Kundu and Basu, 2021). It is possible that the transcriptomic and proteomic profiles reflecting the switching molecular mechanisms involved in low K^+^ induced dormancy or starvation survival of Mab will have a lot in common with dormant Mtb cells obtained under the same conditions, albeit with more pronounced ‘non-culturability’ (Salina et al., 2014a). The question of how well the transcriptomic and proteomic profiles of dormant and starved Mab will align with each other is intriguing.

Here, using a combination of transcriptomic, proteomic, and electron microscopy methods we investigated the strategies of Mab survival during growth arrest and metabolic cessation under potassium deficiency and nutrient starvation.

## 2 Materials and methods

### 2.1 Bacterium and media

*M. abscessus* ATCC 19977^T^ from European Polytechnic School of Lausanne (Lausanne, Switzerland) was stored at −70°C. Starter cultures were initially grown from frozen stocks in Middlebrook 7H9 medium (Himedia, India) with 10% ADS (0.5% BSA, 0.2% dextrose, 0.085% sodium chloride) and 0.05% Tween-80 (Neofroxx GmbH, Germany) for 2–3 days.

For nutrient starvation a mid-log phase culture with OD_600_ = 1.5 was washed twice with 10 mM PBS (8.1 mM Na_2_HPO_4_, 1.5 mM KH_2_PO_4_, 137 mM NaCl and 2.7 mM KCl) containing 0.025% of tyloxapol (Sigma-Aldrich, USA), diluted to OD_600_ = 0.25 in the same buffer with 0.025% of tyloxapol and incubated without shaking at 37°C for 6 weeks.

For low-potassium dormancy starter cultures were initially grown from frozen stocks in Sauton medium containing (per 1L): KH_2_PO_4_, 0.5 g; MgSO_4_·7H_2_O, 1.4 g; L-asparagine, 4 g; glycerol, 60 mL; ferric ammonium citrate, 0.05 g; sodium citrate, 2 g; 1% ZnSO_4_ · 7H_2_O, 0.1 mL; H_2_O, to 1 L; pH 7.0 (adjusted with 1 M NaOH) with 10% ADS (0.5% BSA, 0.2% dextrose, 0.085% sodium chloride) and 0.05% Tween-80 at 37°C with shaking at 200 rpm for 4 days. The grown cultures were inoculated (0.25%) into potassium-free Sauton media in which K^+^ ions (3.7 mM) were equimolarly substituted for Na^+^ ions (Salina et al., 2014b) with addition of 10% ADS and 0.05% Tween-80, and incubated in loose-capped flasks at 37°C with shaking at 200 rpm for 40–45 days.

### 2.2 Viability tests

Tenfold serially diluted cell suspensions were plated in triplicates onto the solid Sauton medium supplemented with 10% ADS in Perti dishes and incubated at 37°C for 6 days, followed by CFU counting. For the MPN assays, the same tenfold serial dilutions were inoculated into liquid Sauton medium supplemented with 10% ADS and 0.05% Tween-80, in 96-well Corning plates. The plates were left to stand at 37°C for 10 days. Wells with visible bacterial growth were counted as positive, and MPN values were calculated with 95% confidence limits using statistical tables designed based on probability histograms (de Man, 1975). MPN values were calculated by counting wells with visible turbidity for a series of at least five serial tenfold dilutions prepared in triplicate. The exact confidence limits, with a minimal probability of 95%, for each MPN value were obtained from the corresponding statistical tables (de Man, 1975).

### 2.3 [^3^H]-Uracil incorporation

Samples of bacterial cultures (1 mL) with *ca* 1×10^8^ cells were incubated with 1 μL of 5.6-^3^H uracil (1 mCi) at 37°C with agitation for 5 h. Two hundred microliters of bacterial cultures were placed in 3 ml 7% ice-cold CCl_3_COOH and incubated on ice for 20 min, followed by filtration through a glass microfiber filter (Whatman, UK). Precipitated cells were washed with 3 ml 7% CCl_3_COOH and 6 ml 96% ethanol. Air-dried filters were placed in 10 ml of scintillation liquid Ultima GoldTM (Perkin Elmer, USA), and the radioactivity incorporation was measured using a LS analyzer (Beckman Instruments, USA) and expressed as counts per minute (cpm).

### 2.4 Transmission electron microscopy

Cells were pelleted at 4000 g for 10 min, washed in sterile mQ water with further centrifugation, fixed in 2.5% glutaraldehyde (w/v) in 0.1 M sodium cacodylate buffer (pH 7.2) for 2.5 h, then post-fixed in 1% (w/v) osmium tetroxide in the same buffer for 12 h. The fixed material was embedded, dehydrated, and polymerized in capsules according to the previously described protocol (Salina et al., 2024). Thin sections were prepared using an Ultrotome III (LKB-Produkter, Sweden), mounted on Formvar-coated copper grids (Jeol, Tokyo, Japan), stained with aqueous 3% uranyl acetate followed by 3.5% lead citrate for 20 min at 37°C, and air-dried for 24 h. Specimens were examined under a JEM-1400 electron microscope (Jeol, Japan).

### 2.5 TEM with energy dispersive X-ray spectroscopy

Pelleted cells were washed with distilled water, dropped in 5–10-μL aliquots onto Formvar-coated and carbon-reinforced copper grids, and air-dried for 12 h. Specimens were subjected to EDX spectroscopy analysis in the TEM mode with the recording of chemical element maps for the whole or selected fields or point spectra using a JEM-1400 microscope (Jeol, Japan) equipped with an energy dispersive X-ray analysis system (EDXA, Inca Energy-350, Oxford Instruments, UK), operating at an accelerating voltage of 80 keV (tilt angle, 15°). The examination procedure was based on selection of a TEM image and EDX spectroscopy to chemical elements in the whole image, or the region of interest with mapping of all or optionally selected elements using Aztec software (Oxford Instruments, UK). A map for each element was automatically marked with different colors. Electron microscopy studies were performed in the UNIQEM Collection Core Facility.

### 2.6 Analysis of images

Individual cells (182 – 226) on appropriate thin section TEM images were assigned to specific morphological types with further calculation of their total occurrence relative to all intact cells for each control or experimental group. The presence of chemical elements in cells for unfixed specimens was demonstrated by comparing elemental maps with the same TEM image recorded prior to X-ray probing. Individual cells (168 – 216) for the control and K^+^-free conditions were grouped to categories of normal (N), depleted (D) and subzero (sZ) intracellular potassium levels based on the relative K content (%) in total map spectra or point spectra through a cell. To approximate the total potassium abundance, we proposed to use a parameter I _K_^+^ = C _K_/S × N_cells_, where C_K_ is the relative K^+^ content (%) in the total map spectrum of a field; S is the total area (in microns) and N_cells_ is the number of cells in the field. The ratios of intracellular to extracellular potassium content were estimated approximately from the K counts in point spectra through a cell and the nearest cell-free space, or from the counts in spectra for areas contouring single or multiple cells versus the extracellular space.

### 2.7 RNA isolation

Bacterial cultures were rapidly cooled on ice, pelleted upon centrifugation (3700 g, 10 min, +4°C), and disrupted with 0.1 mm zirconia beads using a Bead Beater (BioSpec Products, USA) as previously described (Rustad et al., 2008). After extraction with phenol-chloroform, DNA was removed upon treatment with Turbo DNase (Life Technologies, USA). Then, total RNA was isolated and purified using RNeasy Mini Kit (Qiagen, The Netherlands) according to the manufacturer’s protocol. RNA was quantified spectrophotometrically using a NanoDropOne (Thermo Fisher Scientific, USA) and checked for the integrity upon electrophoretic separation in 1% agarose gels.

### 2.8 RNA-seq and data analysis

RNA samples were depleted of 16S and 23S rRNA using the NEBNext^®^ rRNA Depletion Kit (Bacteria) (NEB, USA). Sequencing libraries were generated from the ribosomal transcript-depleted RNA using the NEBNext^®^ Ultra™ II Directional RNA Library Prep Kit for Illumina according to the manufacturer’s protocol. Sequencing was performed on an Illumina NovaSeq 6000 (Illumina Inc, USA) in paired-end mode as 150 nt long reads.

After a quality control evaluation using FastQC (Andrews, 2010), the reads were mapped to the *M. abscessus* reference genome (CU458896.1, http://www.ncbi.nlm.nih.gov/) with Bowtie2 (Langmead and Salzberg, 2012); the alignment was performed with the “-local” and “-dovetail” options. Calculation of the mapped fragments for all genes was performed with the feature Counts program from the Subread package (Liao et al., 2014). Only unambiguously mapped non-chimeric fragments were used in subsequent analysis.

We identified differentially expressed genes (DEGs) using the DESeq2 software package (Love et al., 2014) according to the following criteria: adjusted *p*-value < 0.01 and |log_2_ fold change (log_2_ FC)| value ≥ 2.0. DEGs were then assigned to the functional categories using the EggNOG Database (Huerta-Cepas et al., 2016). Gene set enrichment analysis (GSEA) was performed using GSEA software (Mootha et al., 2003; Subramanian et al., 2005) to identify significantly enriched or attenuated functional pathways. All RNA-seq data were deposited in the GEO repository under the accession number GSE293824. Pearson correlation coefficients between RNA-seq and proteome log2 fold changes were calculated for genes with statistically significant changes in both datasets. Genes with missing values were excluded.

### 2.9 Quantitative real-time PCR

Total RNA (0.1 µg per each sample) was converted to cDNA with random 9-mer primers and M-MuLV reverse transcriptase (Termo Fisher Scientific, USA). Quantitative PCR was performed using qPCRmix-HS SYBR (Evrogen, Russia) and the Real-time CFX96 Touch Cycler (BioRad, USA) under the cycling conditions: 95°C for 10 s, 60°C for 10 s, 72°C for 10 s, repeat 40 times, primers are listed in **Supplementary Table 1**. In the end of amplification, the dissociation curves were plotted to confirm the specificity of the PCR product. All real-time experiments were repeated in triplicate. The results were normalized against the 16S rRNA gene.

### 2.10 Sample preparation for proteomics

Cells from the same cultures, used for RNA isolation, were pelleted upon centrifugation (10 min, 3700 g), resuspended in hot lysis buffer (100 mM Tris-HCl, pH 8.5, 1% SDS, 10 mM DTT), heated at 95°C for 15 min and disrupted with 0.1 mm zirconia beads in a Bead Beater (BioSpec Products, USA). The lysate was centrifuged at 12,000 rpm 4°C for 15 min and filtered through a 20-μm filter. The protein concentration was determined using the micro BCA protein assay kit (Thermo Fisher Scientific, USA). Aliquots containing 50 mg protein were diluted to 1 mg/mL with lysis buffer, and Tris (2-carboxyethyl)phosphine (TCEP) and chloroacetamide (CAA) were added at the final concentrations of 10 and 20 mM for cysteine reduction and alkylation, respectively, performed by heating at 80°C for 10 min. Proteins were precipitated with five volumes of acetone at −20°C overnight; pellets were washed twice with acetone, resuspended in 50 mL of 100 mM Tris pH 8.5, 1% (*w*/*v*) SDS by sonication, and treated with trypsin (Promega, USA) added at the ratio 1/100 (*w/w*, trypsin to protein) for 2 h at 37°C. Then, the second trypsin portion 1/100 *w*/*w* was added, and the samples were incubated overnight at 37°C. Proteolysis was stopped by adding 1% TFA, and precipitated SDS was removed by centrifugation.

### 2.11 Liquid chromatography with tandem mass spectrometry

LC-MS analysis was carried out in an Ultimate 3000 RSLCnano HPLC system connected to a Q Exactive Plus mass spectrometer (TermoFisher Scientific, USA). Protein samples were loaded directly without solid-phase extraction into a trap column (20 × 0.1 mm) packed with Inertsil ODS3 3-mm sorbent (GLSciences, Japan) in the loading buffer (2% acetonitrile, 98% H_2_O, 0.1% trifluoroacetic acid) at the flow rate of 10 mL/min and separated in a fused silica column (500 × 0.1 mm) packed with Reprosil PUR C18AQ 1.9 (Dr. Maisch GmbH, Germany) into the emitter prepared with P2000 Laser Puller (Sutter Instrument Co., USA) (Kovalchuk et al., 2019).

Samples were eluted with a linear gradient of solvent A (0.1% formic acid in water) and solvent B (80% acetonitrile, 19.9% H_2_O, 0.1% formic acid) from 4 to 36% of solvent B over 1 h at 0.44 mL/min at room temperature. MS data were collected in the data-dependent acquisition mode. MS1 parameters were as follows: resolution, 70K; scan range, 350–2000; max injection time, 50 ms; automatic gain control target (AGC), 3 × 10^6^. Ions were isolated with a 1.4 m/z window and 0.2 m/z offset targeting 10 highest-intensity peaks with +2 to +6 charge and 8 × 10^3^ minimum AGC; peptide match was set to preferred, isotope exclusion enabled, and dynamic exclusion set to 40 s. MS2 fragmentation was carried out in the higher-energy collision dissociation mode at 17,5K resolution with 27% normalized collision energy. Ions were accumulated for a maximum of 45 ms with target AGC of 1 × 10^5^.

Each sample was analyzed in three biological replicates. MS raw files were analyzed using PEAKS Studio 8.5 (Bioinformatics Solutions Inc., Canada) (Ma et al., 2003) and peak lists were searched against UniProtKB/TrEMBLE FASTA (canonical and isoform; version of November 2023) for *M. abscessus* ATCC 19977 with methionine oxidation and asparagine and glutamine deamidation as variable modifications. False discovery rate was set to 0.01 for peptide-spectrum matches and determined by searching a reverse database. Enzyme specificity was set to trypsin in the database search. Peptide identification was performed with an allowed initial precursor mass deviation up to 10 ppm and an allowed fragment mass deviation of 0.05 Da. The MS proteomics data have been deposited to the ProteomeXchange Consortium via the PRIDE partner repository (Perez-Riverol et al., 2019) with the dataset identifier PXD065271.

### 2.12 Statistics

Experiments were conducted in three biological replications at least. Statistical analysis was performed using Microsoft^®^ Office^®^ Excel 2016 MSO (16.0.4639.1000). The data were expressed as the mean ± standard deviation. Data were analyzed using Student’s unpaired *t*-test; *p* < 0.05 was considered statistically significant.

## 3 Results

### 3.1 Dormant and starved Mab as models for transcriptomic and proteomic studies

For this study, we reproduced the model of Mab dormancy acquired through prolonged incubations of cultures grown in Sauton-based potassium-free medium (Mulyukin et al., 2023) and employed the model of nutrient starvation in PBS. The physiological and morphological features of the dormant and starved mycobacteria were compared to those of growing mid-log cultures in the complete Middlebrook 7H9 medium and are described below.

#### 3.1.1 Viability and deceleration of metabolic activity

**Figure 1:**
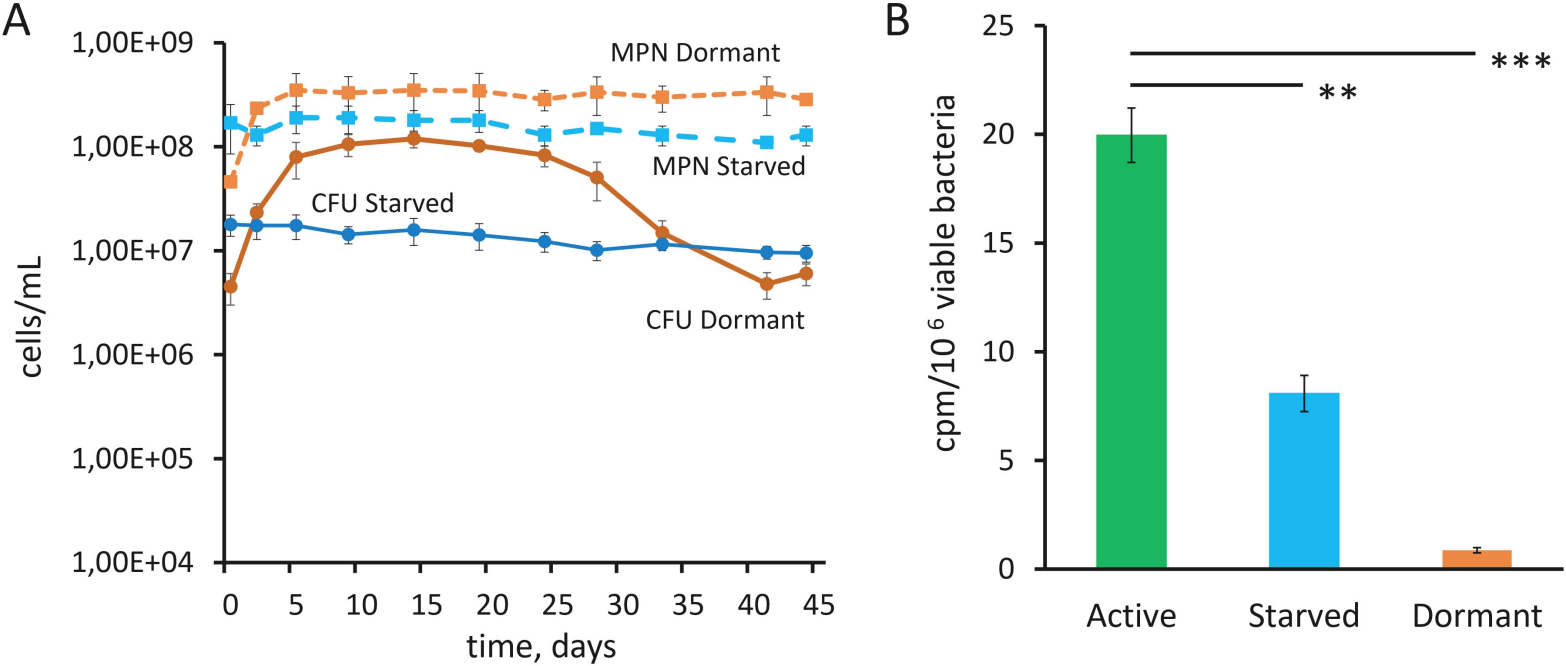
Viability and transcriptional activity of starved and dormant Mab cells. **(A)** CFU and MPN values of Mab cultures under nutrient starvation in PBS and prolonged incubation in K^+^-free medium. The experiment was repeated three times with similar results, a typical experiment is presented. **(B)** Incorporation of [^3^H]-uracil into 3-day active growing mid-log, 14-day PBS-starved and post-stationary 41-day-old dormant Mab cells in K^+^-free medium expressed as counts per minute (cpm) and normalized to the viable cell numbers, corresponding total amount of viable bacteria. The experiments were performed independently twice in triplicates; the mean values and standard deviations are shown here: **p<0.01, ***p<0.001, unpaired *t*-test.

The stationary phase of Mab cultures in K^+^-free medium was reached by day 5, and the further development of already-grown populations occurred without a decline in the number of viable cells capable of growing in fresh complete (with K^+^) Sauton liquid medium as judged by MPN values (**Figure 1A**). Starting on day 28, K^+^-deprived cultures entered a state of transient ‘non-culturability’ with a significant decline of colony-forming ability, as evidenced by CFU dynamics at constant MPN levels (**Figure 1A**). From 41 to 44 days of incubation, over 98% of the Mab population could not be enumerated upon CFU counting though they were detectable using MPN assays (**Figure 1A**). Starved Mab suspensions, prepared by pelleting cells (10 min, 4000 g) from mid-log cultures, followed by washing and resuspending in PBS, exhibited nearly unchanged CFU and MPN titers during incubation for up to 6 weeks (**Figure 1A**). The transition to a non-culturable state did not occur in the starved Mab suspensions judging from that both CFU and MPN counts remained within the same order of magnitude (**Figure 1A**). Mab cells incubated in K^+^-free medium over prolonged incubation period (41 days) were used as the dormant model. This time point was selected on the basis of the substantial loss colony formation (CFU counts) yet the sustained viability (MPN counts) (**Figure 1A**). The other physiological state is represented by Mab cells, which were incubated in PBS for 14 days and are used as the starvation model. Both CFU and MPN values remained constant over a 45-day period (**Figure 1A**), and the selection of the time point was not crucial in terms of viability maintenance by starved Mab. Both these models of non-culturability of Mab were subjected to further analysis of metabolic activity, cell morphology, and transcriptomic and proteomic profiles.

Normalized ^3^H‐uracil incorporation values (radioactivity counts per minute, cpm, per the viable cell numbers) were statistically significantly lower by more than twenty times in samples of post-stationary 41-day-old dormant Mab cells in K^+^-free medium than in samples from mid-log cultures (3 days) in the complete Middlebrook 7H9 medium (**Figure 1B**). These results suggest a profound decrease in transcriptional activity in dormant Mab cells under the conditions used, though not to undetectable levels. Starved Mab cells (14 days) displayed a transcription activity at the ~40% level of actively growing cultures, as inferred based on the corresponding values of ^3^H‐uracil incorporation (**Figure 1B**).

#### 3.1.2 Transmission electron microscopy imaging

##### 3.1.2.1 Active cultures

**Figure 2:**
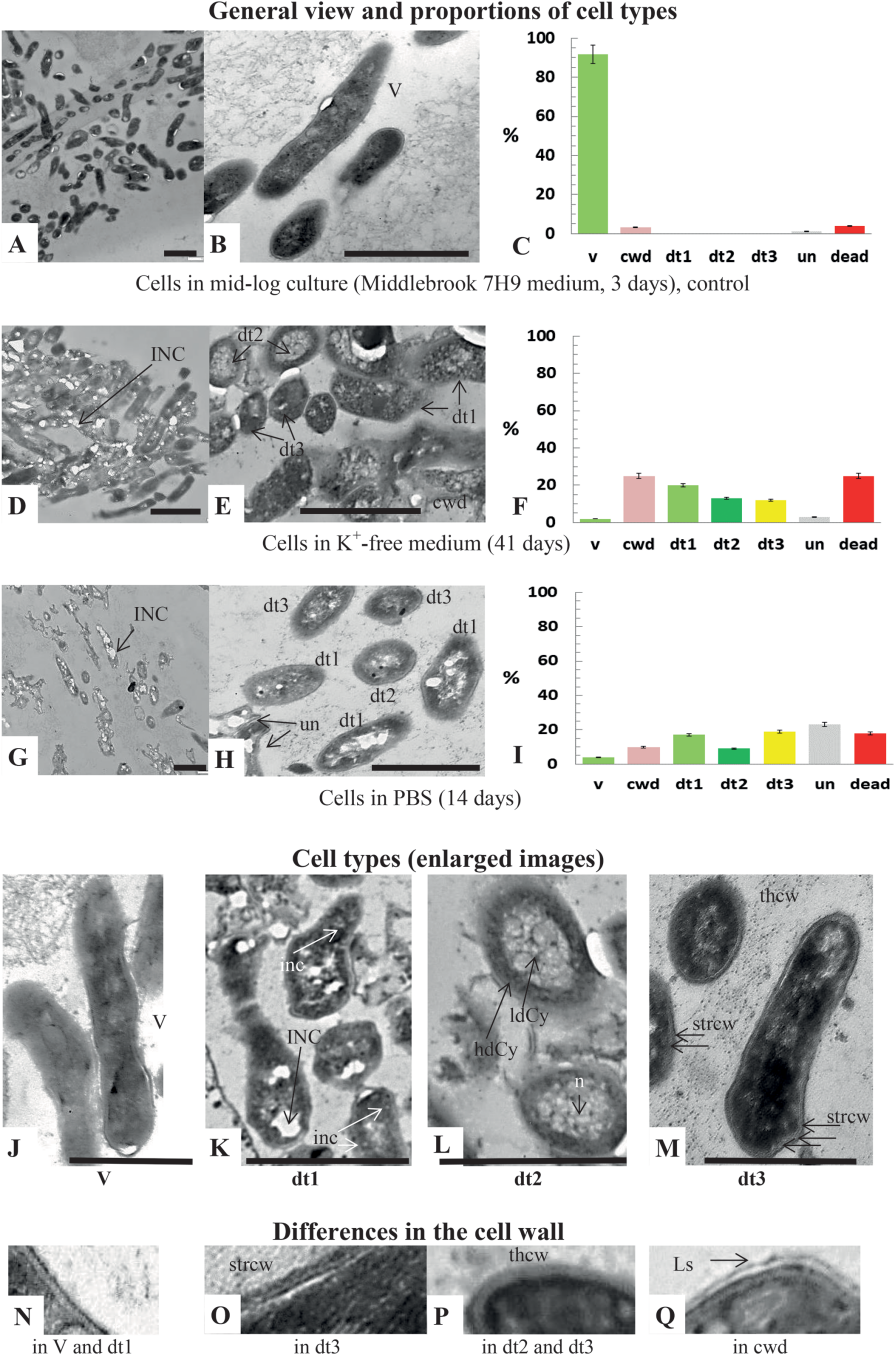
Thin section TEM images of *M. abscessus* cells in mid-logarithmic cultures (3 days), dormant K^+^-depleted cultures (41 days), and starved suspensions in PBS (14 days). General views **(A, B, D, E, G, H)**, enlarged images of the different cell types **(J, K, L, M)**, and magnified fragments of cell envelopes **(N–Q)** are shown. Bars: **(A, B, D, E, G, H)** = 1 µm; **(J–M)** = 500 nm. The percentage of different cells types **(C, F, I)** was calculated based on the examinations of 182–226 cells. Designations for cell types: V, vegetative cells; dt1, cells with the granules and electron-transparent inclusions; dt2, cells exhibiting heterogeneous cytoplasm, characterized by an electron-dense periphery and a low-density central part; dt3, cells with altered cell walls and the dense, homogeneous cytoplasm; un, uncommon morphological type; cwd, cell-wall-deficient cells. Comments and descriptions can be found in the section 3.1.2. Other designations: inc and INC, small and large transparent or low-density inclusions, respectively; hdCy and ldCy, highly dense and low-density regions in the cytoplasm, respectively; strcw and thcw, stratified and thickened cell walls, respectively; n, nucleoid; Ls, a loosened mycolic acid layer in cell-wall deficient cells. Individual layers in stratified cell walls are demarcated with arrows (image **M**).

Mab cells that possessed the intact cell wall and cytoplasmic membrane, and finely grained cytoplasm (classified as vegetative cells, **Figures 2A, B** and magnified images **Figures 2J, N**) constituted the predominant (92%) cell type V in the control mid-log cultures grown in the complete Middlebrook 7H9 medium. Dead and cell-wall deficient cells comprised a minor fraction, amounting to no more than 4% of total cells (**Figure 2C**).

##### 3.1.2.2 General features of dormant and starved cultures

Mab cells in 41-day-old cultures in K^+^-free medium (**Figures 2D, E**) or in cultures starved for 14 days (**Figures 2G, H**) exhibited differences in morphology compared to vegetative cells (**Figures 2A, B**). The substantial fraction of intact cells in the aged cultures exhibited the morphological traits of dormant Mab, characterized by the absence of cell division, the preservation of subcellular structures, and less or more pronounced changes in the cell envelopes and/or the cytoplasm. Cells containing conspicuous electron-transparent inclusions in the cytoplasm constituted approximately 20% and 28% in dormant and starved cultures, respectively (**Figures 2F, I**) and were rarely observed (~ 1%) among actively growing Mab cells (**Figure 2C**).

##### 3.1.2.3 Cell types

Dormant cells were classified into three distinct morphological types (dt1, dt2, dt3) (**Figures 2K–M**), which collectively constituted approximately half (46 – 48%) of the cell population that was subjected to K^+^-deprivation or starvation (**Figures 2F, I**). The dt1 type was distinguished by the presence of electron-transparent inclusions of various sizes and numerous granules of low density (**Figure 2K**). The type dt2 of cells was characterized by the heterogeneous cytoplasm with an electron-dense periphery and a low-density central part (**Figure 2L**). The type 3 encompassed cells exhibiting the stratified cell wall composed of alternating layers with varying density (**Figures 2M, O**) or the thickened and homogenous cell envelope (**Figure 2P**) and homogenous cytoplasm. The profile of cellular envelopes of the d3 type cells was found to differ from that of vegetative cells (**Figure 2N**). Cells that exhibited a curved or angular shape and inclusions (**Figure 2H**) were classified under to the category “un”, which designates an uncommon type. Consequently, the morphological diversity of Mab cells was intrinsic to the dormant and starved Mab cultures in contrast to actively growing populations.

##### 3.1.2.4 Minor differences

Intact curved cells belonging to the un type (**Figure 2H**) accounted for approximately 23% in the starved populations (**Figure 2I**) and were almost absent in the control and aged K^+^-sequestered cultures (**Figures 2C, F**). The features of cell wall deficiency, including disruption of the outer mycolic acid layer (**Figure 2Q**), were more prevalent (~25%) for Mab under long potassium deficiency stress than in the starved (10%) and control (2.5%) cultures (**Figures 2C, F, I**, respectively). Notwithstanding these insignificant differences, dormant and starved Mab populations exhibited comparable morphologies.

#### 3.1.3 Biogenic elements and cationic homeostasis

**Figure 3:**
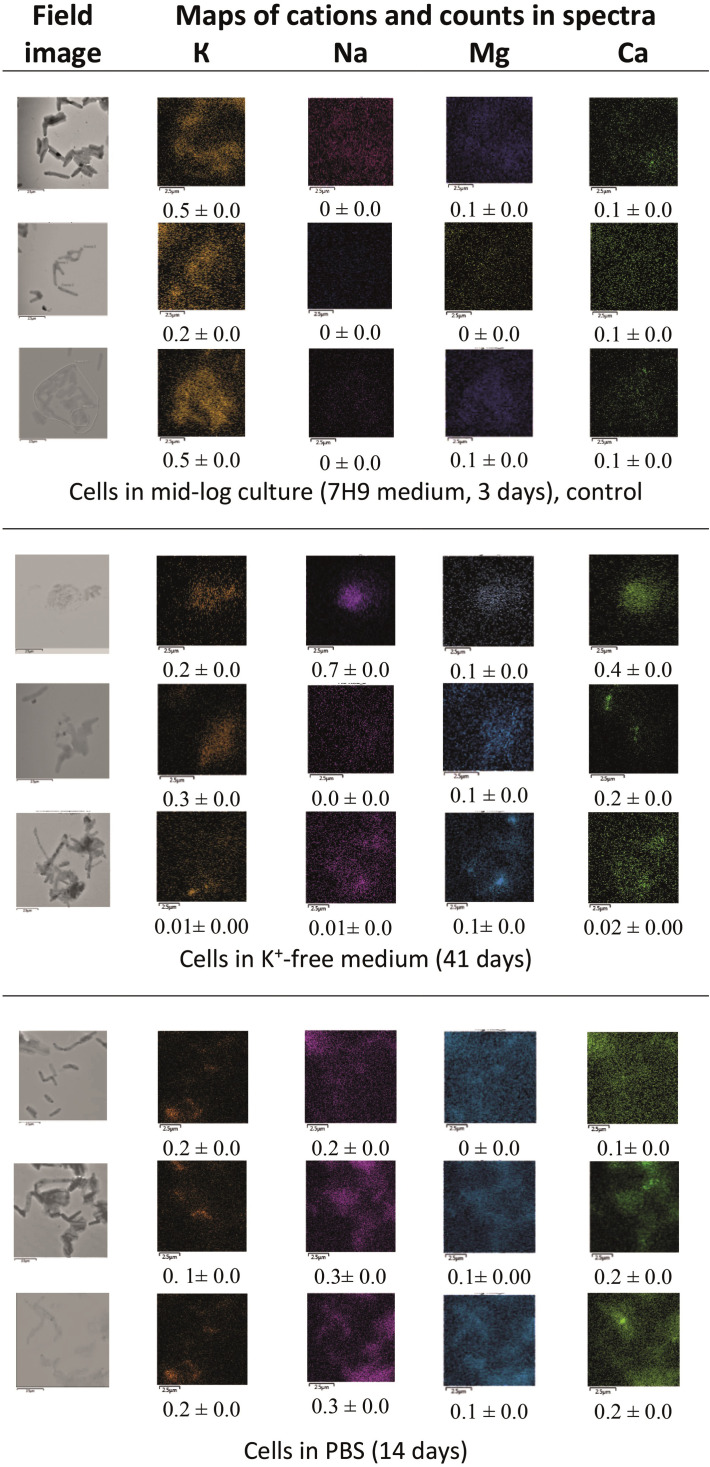
TEM-EDX analysis: images of fields with *M. abscessus* cells and colored maps of potassium, sodium, magnesium, and calcium. Bars, 2.5 µm.

Qualitative TEM-EDX spectroscopy analysis with mapping of carbon, oxygen, phosphorus, sulfur, nitrogen, and cations in fields with mycobacteria and cell-free space showed that the majority of cells maintain pools of biogenic elements in both the active and aged cultures (**Supplementary Figure 1**). Cells in 41-day old K^+^-depleted cultures and 14-day starved suspensions had decreased pools of P, S and N compared to actively growing populations (**Supplementary Figure 1**). Overall, Mab cells subjected to prolonged starvation or potassium deprivation were heterogeneous in the accumulation of monovalent (K and Na) and bivalent (Mg and Ca) cations. Some cells with depleted K^+^ levels in the both aged cultures contained higher amounts of the other cations, especially calcium, while actively growing mycobacteria with well detectable potassium levels contained low amounts of Na, Mg, Ca (**Figure 3**; **Supplementary Figure 1**).

Based on the EDX spectra, we approximated the proportions of cells with the normal (N), deficient (D), and subzero (sZ) levels of potassium in the control mid-log cultures and long-stored K^+^-deprived populations for which initial sources of potassium were the inoculated cells and probably admixtures in reagents. In the control cultures, the cells with the normal K^+^ constituted 87%, and potassium-deficient and potassium-free mycobacteria shared smaller fractions (8% and 5%, respectively) (**Supplementary Table 2**). The potassium abundance index I_K+_, which we derived from EDX spectral data, varied from 0.15 ×10^–3^ to 0.29 ×10^–3^ in normalized units of the relative K^+^ content (%/µm^2^×N_cells_) for mid-log cultures growing without potassium deficiency. The ratio of intracellular to extracellular potassium (K^+int^/K^+extr^) was in a rather narrow range, from 1 to15 (**Supplementary Table 2**).

Under long-term K^+^ deprivation, cells with reduced and subzero K^+^ levels (35% and 36%) were dominant over cells with the normal or near normal potassium pool, as judged from differential counting cells in all examined fields and can be visualized from elemental maps (**Figure 3**; **Supplementary Figure 1**, **Supplementary Table 2**). It is noteworthy that the mycobacterial culture under prolonged potassium depletion stress appeared heterogeneous at both the single cell and multicellular levels. There were cells with the similar I_K+_, and K^+int^/K^+extr^ parameters as in the case of no-deficiency growth (subpopulation 1); small clumps with the fewer I_K+_ and higher K^+int^/K^+extr^ than for those active culture (subpopulation 2), and clumps experiencing severe potassium deficiency (subpopulation 3) in which few cells only were able to maintain the potassium homeostasis (**Supplementary Table 2**).

Post-stationary Mab cells grown in K^+^-free medium, which remained viable over prolonged incubation period (41 days) despite losing the colony-forming ability, and Mab cells, incubated in PBS for 14 days, showed significantly reduced metabolic activity and changed morphology. For brevity, we will use below the designations ‘active’, ‘dormant’ and ‘starved’ cells without detailing of the growth stage, media, and incubation time.

### 3.2 Transcriptomic profiles of dormant and starved Mab

#### 3.2.1 General description

RNA-seq data from three replicates of active, dormant, and starved Mab cultures were analyzed to identify up- or down-regulated genes. The analysis showed the less percentage of mapped coding sequences (CDS) in dormant cells (average 44.40%) and starved cells (average 47.88%) than in active bacteria (average 67.56%) (**Supplementary Table 3**). Differentially expressed genes (DEGs) were defined as genes whose expression changed by more than fourfold (|Log_2_FC| > 2, p.adj ≤ 0.01) in dormant or starved Mab compared to active cells. A total of 668 DEGs were identified in dormant Mab, of which 527 were up-regulated and 141 were down-regulated (**Supplementary Table 4**). In starved Mab, 692 DEGs were detected, 436 were up-regulated and 256 were down-regulated (**Supplementary Table 4**).

**Figure 4:**
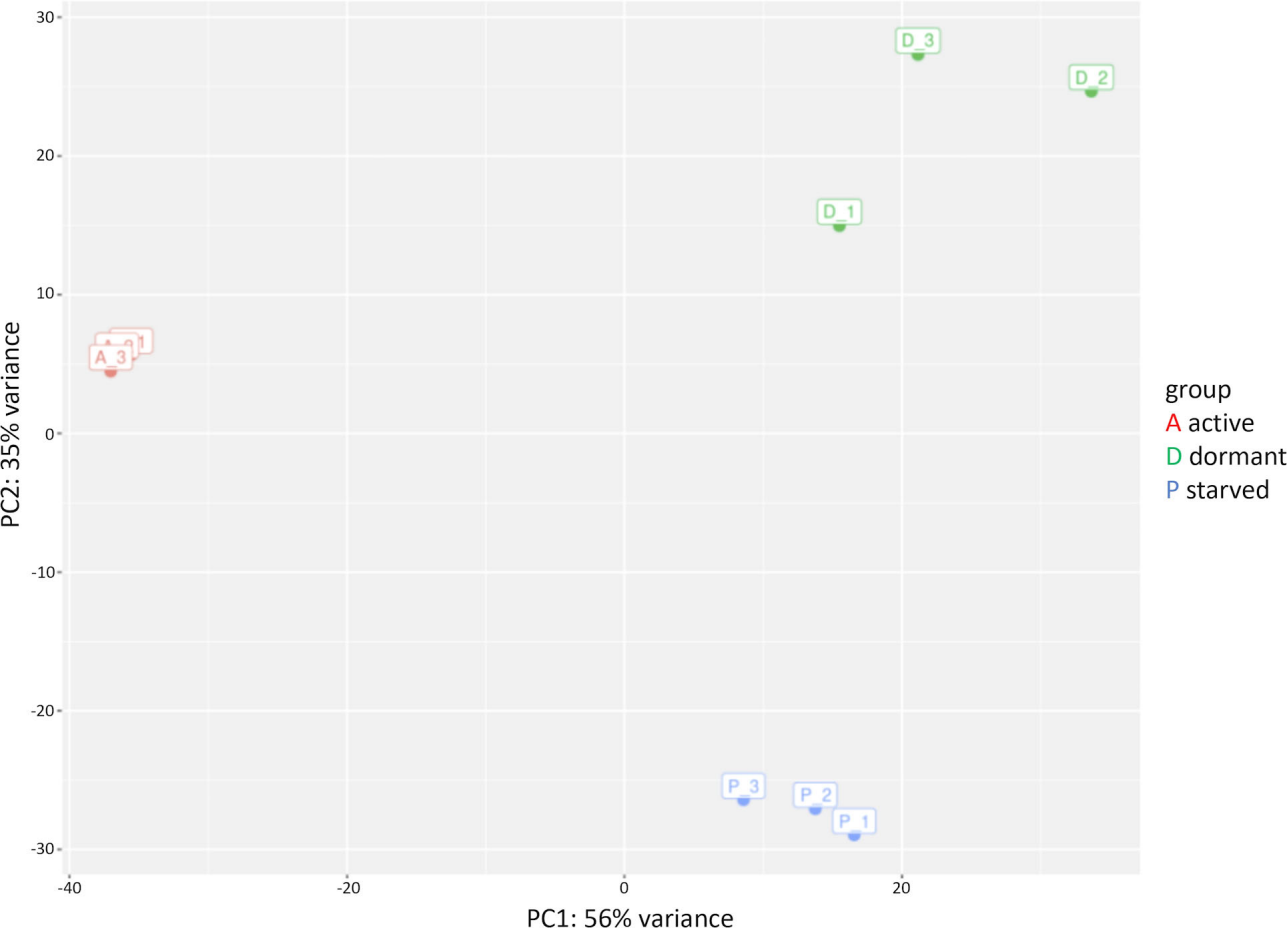
Principal component analysis (PCA) of 3-day active mid-log (A), 41-day dormant (D), 14-day PBS-starved (P) Mab cultures.

**Figure 5:**
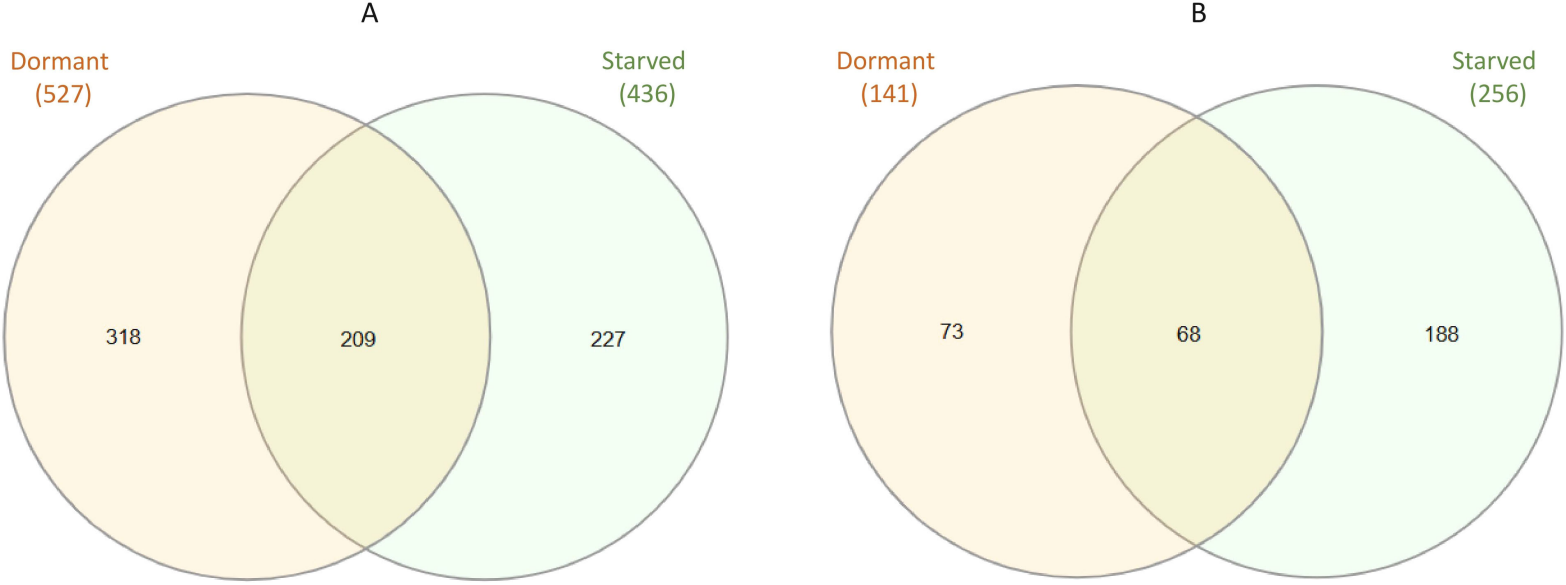
The Venn diagram of transcriptomic responses in dormant and starved Mab in comparison to active grown mid-log bacteria grown in balanced medium. **(A)** Up-regulated genes. **(B)** Down-regulated genes. (|Log_2_FC| > 2, p.adj ≤ 0.01.

Principal component analysis (PCA) confirmed the technical quality of the transcriptomes and revealed unique transcriptional clusters for active, dormant, and starved Mab (**Figure 4**). The transcriptomic profiles of dormant and starved Mab displayed a partial overlapping in DEGs, as shown in the Venn diagrams (**Figure 5**). Of the up-regulated DEGs, a total of 209 were common, while 318 and 227 were specific to dormant and starved mycobacteria, respectively (**Figure 5**). Of the repressed genes, 68 were common, and while 73 and 188 appeared specific to the corresponding physiological states (**Figure 5**). The expression level of some DEGs was confirmed by qPCR (**Supplementary Figure 2**).

#### 3.2.2 Functional categories of DEGs

**Figure 6:**
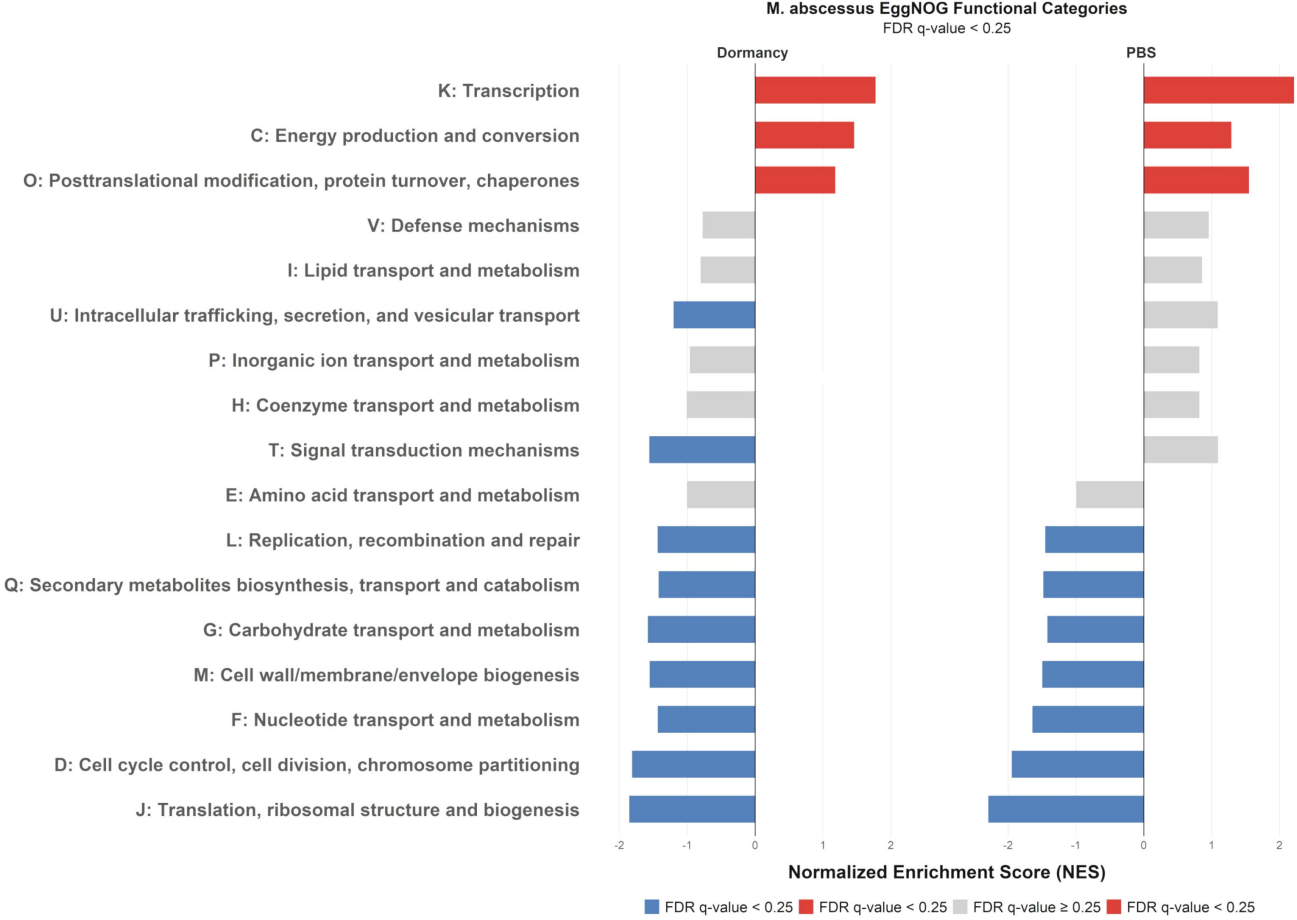
Gene set enrichment analysis (GSEA) results comparing dormant and starved versus active grown mid-log Mab. The EggNOG functional categories were analyzed to identify differentially enriched gene sets. Bars in red indicate significant enrichment under potassium deficiency and nutrient starvation (FDR q-value < 0.25), bars in blue show significant down-regulation in these conditions (FDR q-value < 0.25), and bars in gray represent non-significant gene sets (FDR q-value ≥ 0.25).

In order to gain some insight into general mechanisms involved in the maintenance of bacterial viability over prolonged pressure of the growth-unsupportive conditions, the DEGs were grouped into the functional categories in accordance with the EggNOG Database (Huerta-Cepas et al., 2016) Up-regulated genes for both dormant and starved Mab cultures showed overlapped enrichment in three key functional EggNOG categories: K (transcription), C (energy production and conversion) and O (post-translational modification, protein turnover, chaperones) as shown in (**Figure 6**).

The down-regulated genes in the Mab’s models fell into a broader range of the categories: J (translation, ribosomal structure and biogenesis), D (cell cycle control, cell division, chromosome partitioning), G (carbohydrate transport and metabolism), M (cell wall/membrane/envelope biogenesis), F (nucleotide transport and metabolism), L (replication, recombination and repair), Q (secondary metabolites biosynthesis, transport and catabolism). Specifically, the down-regulated genes in dormant (but not in starved Mab) were found in the T (signal transduction) and U (intracellular trafficking, secretion, and vesicular transport) categories.

#### 3.2.3 Activated and suppressed KEGG pathways

Gene Set Enrichment Analysis (GSEA) data demonstrated that dormant Mab showed significant enrichment in a total pool of transcripts which are predicted to encode enzymes involved in steroid degradation and lipoic acid metabolism (**Supplementary Figure 3**). Similarly, starved cells also contained elevated levels of these transcripts, although the pathway enrichment did not reach statistical significance (FDR q-value ≥ 0.25) (**Supplementary Figure 3**).

**Figure 7:**
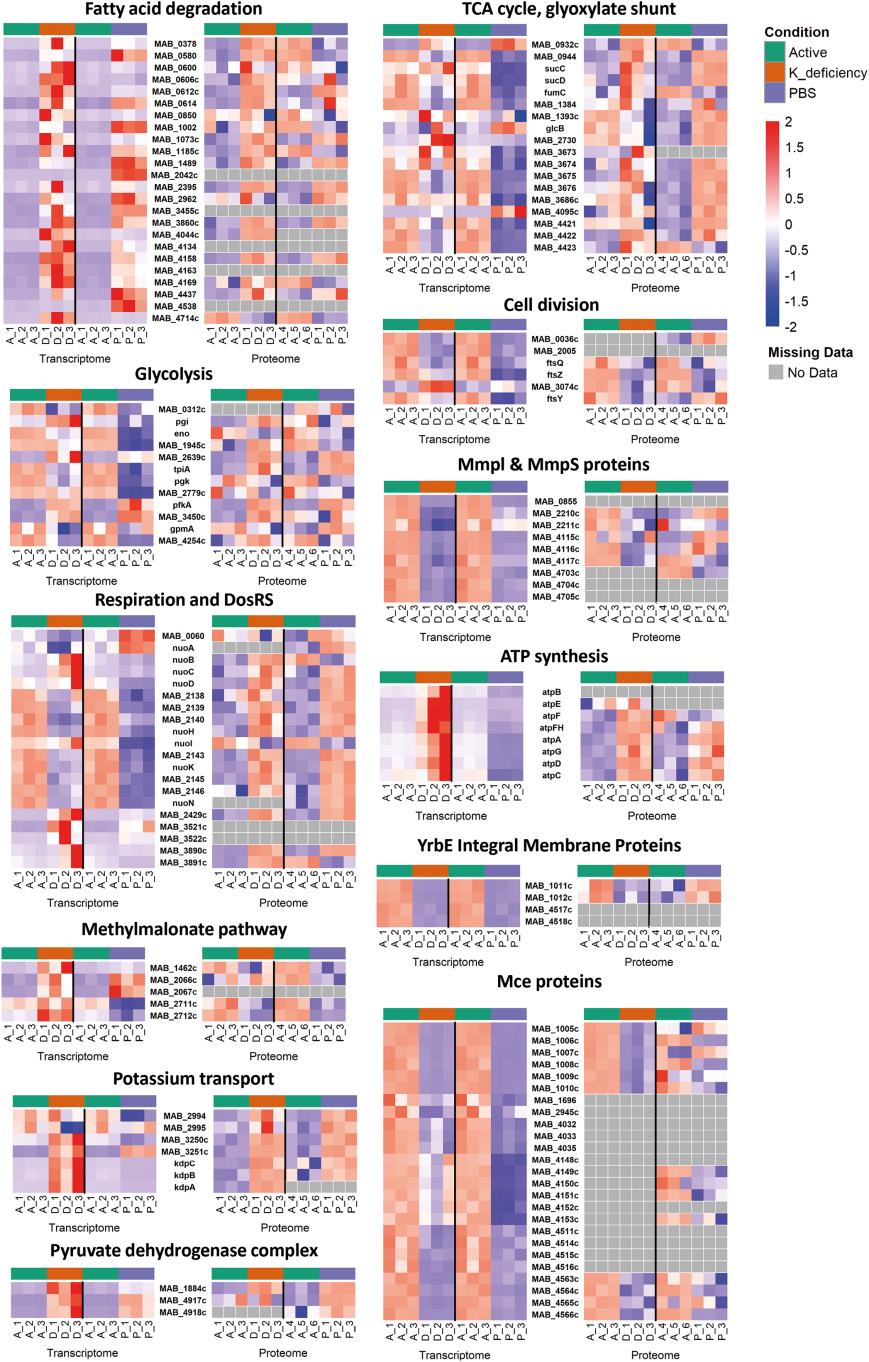
Representation of normalized expression levels of selected genes and proteins involved in key metabolic pathways in active mid-log (A), dormant (D), and PBS-starved (P) Mab. Transcriptomic data (left side of each panel) and proteomic data (right side of each panel) are shown for comparison. Gene expression data were normalized using DESeq2, while protein abundance data were analyzed using appropriate proteomics workflows. Expression values were normalized to z-scores across samples and displayed according to the color code, where red indicates higher expression and blue indicates lower expression relative to the mean. In RNA-seq studies A_1, A_2, A_3 are control samples both for D_1, D_2, D_3 and P_1, P_2, P_3 conditions; in proteomic studies A_1, A_2, A_3 are control samples for D_1, D_2, D_3, and A_4, A_5, A_6 are control samples for P_1, P_2, P_3 conditions.

Up-regulated genes included those for lipases, fatty acid-CoA ligases, lipid-transfer protein, acyl-CoA dehydrogenase, acetyl-CoA transferase, enoyl-CoA hydratase, acyl-CoA thiolase, acetyl-CoA acetyltransferases, enoyl-CoA hydratases, providing the accumulation of acetyl-CoA (and propionyl-CoA) and were present at higher levels in dormant and, to a lesser extent, in starved mycobacteria, than in active cells (**Figure 7**; **Supplementary Table 4**). KEGG pathways of nitrogen metabolism and 2-oxocarboxylic acid metabolism were enriched in dormant and to a less extent in starved Mab (**Supplementary Figure 3**). Transcripts, corresponding to the pyruvate dehydrogenase complex, which is responsible for producing acetyl-CoA from pyruvate, as well as the enzymes involved in the methylmalonyl pathway, which is crucial for the degradation of odd-chain fatty acids showed increased abundance both in dormant and starved Mab (**Figure 7**; **Supplementary Table 4**). The both studied NRS had the increased levels of transcripts for isocitrate lyase MAB_4095c, the key enzyme of glyoxilate shunt and citrate synthase MAB_0932c, the key enzyme of TCA cycle. The other TCA cycle’s enzymes were mostly repressed (in starved bacteria) or unchanged (in dormant bacteria) (**Figure 7**; **Supplementary Table 4**). Upregulation of nitrite reductase *nirBD* (MAB_3521c and MAB_3522c, respectively) together with non-proton-pumping type II NADH dehydrogenase *ndh* (MAB_2429c) was found in dormant Mab only with concomitant repression of proton-pumping type I NADH dehydrogenase (**Figure 7**; **Supplementary Table 4**).

Both dormant and starved Mab cells had repressed or unchanged pathways of the utilization of simple sugar-based carbon source via glycolysis and pentose phosphate **Figure 7**; **Supplementary Table 4**). On the whole, growth-related, energy-producing and biosynthetic processes were expectedly attenuated in NRS, highlighting a shift from active proliferation to resource conservation. Cell division proteins, including RodA (MAB_0036c) and proteins of Fts family, were found to be down-regulated in dormant and starved bacteria, confirming the growth arrest in NRS (**Figure 7**; **Supplementary Table 4**). Some proteins of MmpL and MmpS family associated with mycobacterial virulence, as well as proteins of YrbE family, participating in ‘host-pathogen’ interaction, and lipid-transporting proteins of Mce proteins, modulating host cell signaling, were repressed in dormant and starved Mab (**Figure 7**; **Supplementary Table 4**).

The inducible potassium-transporting Kdp system was up-regulated in dormant Mab only, while the major constitutive potassium transporter consisting of two Trk proteins, CeoB and CeoC, was unchanged in the both NRS types (**Figure 7**; **Supplementary Table 4**). It is noteworthy that the dormancy survival regulator *dosR* (MAB_3891c), its counterpart sensor kinase *dosS* (MAB_3890c), and ATP synthase’s subunits *atpA-H* were activated only in dormant cells under potassium deficiency (**Figure 7**; **Supplementary Table 4**).

### 3.3 Proteomic profiles of dormant and starved Mab

Proteomic datasets for active, dormant, and starved Mab were produced and analyzed to reveal different protein expression. Similar to DEGs, differentially expressed proteins (DEPs) were proteins whose abundance differed by |Log_2_FC| > 2 (q-value ≤ 0.05) from active cells. Totally, 557 DEPs were identified in dormant Mab; 228 of them were up-regulated and 329 were down-regulated. Starved Mab showed 833 DEPs, 266 up-regulated and 567 down-regulated (**Supplementary Table 5**).

#### 3.3.1 Up- or down-regulation in both proteome and transcriptome

Different regulation of proteins showed a strong positive correlation (r = 0.656) with transcriptomic changes in dormant Mab, in contrast to much weaker correlation (r = 0.168) between transcriptome and proteome data for starved Mab (**Supplementary Figure 4**). Consistent with the up-regulation of transcripts, both dormant and starved Mab were enriched in proteins involved in fatty acid degradation and constituting the pyruvate dehydrogenase complex, as well as transcriptional regulatory proteins of various families (**Figure 7**; **Supplementary Tables 5, 6**). Specifically, dormant Mab contained an elevated pool of dormancy regulator DosR (MAB_3891c) (log_2_FC = 2.03) correspondingly to its enhanced transcription (log_2_FC = 3.97). Notably, the activation of the potassium-transporting Kdp system in dormant Mab was evident in both proteome and transcriptome profiles (**Figure 7**; **Supplementary Tables 5, 6**). In dormant Mab, ATP-synthase subunits were upregulated at the both translation and transcription levels **Figure 7**; **Supplementary Tables 5, 6**).

In accordance with the transcriptome profile, dormant and starved Mab exhibit a depletion of proteins implicated in cell division (i.e., of the Fts family); in the synthesis of the cell wall and the cell membrane components, and in a variety of biosynthetic pathways (**Figure 7**; **Supplementary Tables 5, 6**). It is noteworthy that some proteins of MmpL and MmpS family, which are associated with mycobacterial virulence, and lipid-transporting Mce proteins, modulating host-cell signaling, were also reduced in the both NRS types, in concordance with the down-regulation of their transcription.

#### 3.3.2 Overrepresentation in proteome *vs* depletion in transcriptome

The elevated or reduced levels of DEPs corresponded well to the up- or down-regulated transcription of their genes in dormant Mab (**Figure 7**; **Supplementary Table 6**). In starved cells there were a significant proportion of proteins whose expression levels changed in the opposite direction.

Starved Mab cells exhibited enrichment in ATP-synthase, proton-pumping type I NADH dehydrogenase, enzymes of TCA, and demonstrated a depletion of the corresponding transcripts (**Figure 7**; **Supplementary Table 6**). The universal stress protein MAB_2489 was represented well in the proteome (log_2_FC = 4.03) and down-regulated in the transcriptome of starved Mab (log_2_FC = – 4.45), as well as UbiA prenyltransferase MAB_0173 (log_2_FC = 5.73 *vs* –2.61), which is involved in ubiquinone synthesis (**Supplementary Table 6**). The predicted non-ribosomal peptide synthase, MAB_0510c, which can be implicated in the cell response to a lack of ribosomal activity and in an alternative mechanism of assembling essential proteins from non-proteinogenic substrates, was found at high levels in the proteome (log_2_FC = 3.79), oppositely to its transcription (log_2_FC = – 4.45). The DNA-binding histone-like protein, MAB_3292c, which participate in H_2_O_2_ defense and thus contributes to bacterial survival, was present at substantial levels (log_2_FC = 2.56) in starved Mab thought its transcription was suppressed (log_2_FC =–3.39) (**Supplementary Table 6**). It is noteworthy that starved cells had an enriched pool of proteins (oppositely to transcripts) such as arabinosyltransferase, MAB_0189c, (log_2_FC = 2.41 in proteome *vs* log_2_FC = –2.24 in transcriptome) and UDP-galactofuranosyl transferase, MAB_0171, (log_2_FC = 2.14 *vs* log_2_FC = –2.38). These enzymes play a pivotal role in the polymerization of arabinogalactan, the key component of the mycobacterial cell wall, and can be involved in cell wall strengthening during the initial stages of adaptation.

#### 3.3.3 Underrepresentation in proteome *vs* enrichment in transcriptome

Both dormant and starved Mab exhibited a decrease in proteins involved in antioxidant mechanisms, including thioredoxin (MAB_2739c), glutaredoxin (MAB_3994c), deazaflavin-dependent nitroreductase (MAB_2860c), and in the initiation of transcription including factor RbpA (MAB_2208c), sigma-70 factor (MAB_1363), and sigma factor modulator (MAB_2512), though their transcription was increased (**Supplementary Table 6**). Specifically, dormant Mab cells were enriched in a positive regulator of the DosRS system, MAB_2014c, contrastingly to its transcript (log_2_FC = – 2.19 and 2.04, respectively). Starved Mab were depleted in the other predicted DosRS regulators, such as MAB_4426 (log_2_FC = –2.61 and 5.04), MAB_2562c (log_2_FC = –2.50 and 4.36), and MAB_2386 (log_2_FC = –2.05 and 2.38), oppositely to their evident enrichment in the transcriptomic profile (**Supplementary Table 6**).

## 4 Discussion

Evidently, both dormancy under potassium deficiency and starvation in PBS model NRS of Mab have both common and distinctive features. Not only dormant but also starved cells possess the retained viability and the reduced metabolism (**Figure 1**). The results of the TEM examinations provide evidence that the two NRS populations examined under this study exhibit differences in morphology when compared to actively growing Mab, as previously reported (Mulyukin et al., 2023). Furthermore, these populations demonstrated a notable heterogeneity in the structure of cells, a feature that distinguishes them from active cultures. Finally, these NRS populations harbored similar cell types to be essential for adaptation (**Figure 2**). Nevertheless, the observed cell morphologies of Mab (**Figure 2**) appear to differ from surviving Mab populations that were initially grown in rich liquid medium and subjected to harsh antibiotic treatment for prolonged period (Salina et al., 2024). A significant proportion of post-stationary Mab cells, which were incubated for prolonged time in the spent K^+^-free medium, lost the ability to form colonies, adopting a transitional ‘non-culturability’, and required a special condition to resume growth (i.e., the use of liquid medium). Moreover, they showed strongly diminished ^3^H‐uracil incorporation, an indicative of metabolic repression. On the contrary, Mab cells survived in PBS without significant loss of colony-forming ability and exhibited a higher level of metabolic activity than Mab cultures under potassium sequestration (**Figure 1**). Based on these differences, long-stored K^+^-depleted Mab cultures were considered as model of deep dormancy, whereas starved Mab were regarded to be in immature quiescent state.

The potassium deficiency was chosen for this study not only due to its importance for Mab survival under different conditions, including antibiotic treatment (Mulyukin et al., 2023; Salina et al., 2024). Potassium, as an abundant cation, regulates numerous metabolic pathways in various bacteria, including Mtb (Tan, 2021; Chen et al., 2025) and its role in the bacterial response to diverse environmental cues extends beyond controlling osmotic stress (Epstein, 2003; Wood, 2011). Particularly, potassium deficiency can occur in phagosomes due to the operation of a K^+^-efflux pump (Wiese and Seydel, 1996; Amaral et al., 2007). The ability of dormant Mab cells to cope with potassium deficiency by maintaining (or not) very low or near-normal K^+^ levels or by taking up other cations (**Figure 3**; **Supplementary Table 2**) reflects adaptability to changes in its concentrations at particular loci.

The main and new is the finding, that despite two distinctive stimuli to form NRS, both dormant and starved Mab have common changes in their transcriptomic and proteomic signatures. These changes reflect global reprogramming of bacterial metabolism which implies switching off the majority of growth- and biosynthesis-related activities and enhancing some ‘alternative’ pathways, including those involved in utilization of lipids and fatty acids as carbon and energy source, and glyoxylate shunt (**Table 1**, **Figure 7**; **Supplementary Tables 4, 5, 6**). It is noteworthy, that elevated level of protein and/or transcript for isocitrate lyase, nitrate reductase NirBD, and uncoupled non-proton-transporting type II NADH dehydrogenase with the suppression of the proton-transporting type I NADH dehydrogenase operon, especially in dormant Mab, denotes a ‘hypoxic-like’ response though our experimental conditions did not imply oxygen deprivation. The similar modulation pattern was observed for profoundly ‘non-culturable’ Mtb cells formed under the similar conditions (Salina et al., 2014a; Ignatov et al., 2015 BMC). Consequently, a ‘hypoxic-like’ response can be regarded as the general adaptation program for persisting mycobacteria, ensuring their survival in NRS by a ‘lean’ scenario due to suppression of the central metabolic pathways.

**Table 1:** Up- and down regulation of Mab metabolic pathways under various stress conditions.

Condition	Selected upregulated genes and pathways	Selected downregulated genes and pathways	References
Dormancy under low-K^+^ conditions, 41 d	inducible potassium-transporting Kdp system β-oxidation of fatty acids; glyoxylate shunt; pyruvate dehydrogenase complex; methylmalonyl pathway; proton-transporting NADH dehydrogenase type-I; ATP synthase subunits, *dosRS*; oxidative and multiple stress response; heat shock proteins *whiB7*	glycolysis and pentose phosphate pathways; ribosomal proteins; cell division; proton-transporting NADH dehydrogenase type I; MmpL and MmpS proteins, Mce proteins, YrbE proteins mycobactin clusters	This study
Nutrient limitation in PBS, 14 d	β-oxidation of fatty acids; glyoxylate shunt; pyruvate dehydrogenase complex; methylmalonyl pathway oxidative and multiple stress response; heat shock proteins *nirBD*; *whiB7*	glycolysis and pentose phosphate pathways; ribosomal proteins; cell division; ATP synthesis; proton-transporting NADH dehydrogenase type I; TCA cycle MmpL and MmpS proteins, Mce proteins, YrbE proteins mycobactin clusters	This study
NO induced hypoxia, 40 min	β-oxidation of fatty acids; *dosRS*; multiple stress response; heat shock proteins mycobactin clusters; WhiB7 regulon	ribosomal proteins	Miranda-CasoLuengo et al., 2016
Growth in an artificial sputum (synthetic CF sputum medium), 3 h	pyruvate dehydrogenase complex; glyoxylate shunt; proton-transporting NADH dehydrogenase type I WhiB7 regulon	ribosomal proteins Mce proteins, YrbE proteins	Miranda-CasoLuengo et al., 2016
Intracellular survival in murine macrophages, 16 h	β-oxidation of fatty acids; glyoxylate shunt; pyruvate dehydrogenase complex; cholesterol metabolism, methylmalonyl pathway; *dosR;* oxidative and multiple stress response heat shock proteins	glycolysis and pentose phosphate pathways; the mycolate operon;	Dubois et al., 2019
hypoxia 1% O_2_, 5 d	β-oxidation of fatty acids; cholesterol metabolism, *dosRS*; proton-transporting NADH dehydrogenase type I, ATP synthase subunits, Mce proteins	ribosomal proteins; nitrite reduction and extrusion	Simcox et al., 2023
Co^2+^ 625 μM, 2 h	glyoxylate shunt WhiB7 regulon	TCA cycle; glycolysis; proton-transporting NADH dehydrogenase type I; ATP synthase subunits,	Liu et al., 2024
Ni^2+^ 2500 μM, 2 h	glyoxylate shunt WhiB7 regulon	glycolysis; proton-transporting NADH dehydrogenase type I; ATP synthase subunits	Liu et al., 2024

As expected, a divergence in the transcriptomic and proteomic profiles between the two NRS Mab models examined in this study was observed. Firstly, the activation of the inducible potassium-transporting Kdp system was inherent in dormant Mab obtained under prolonged potassium sequestration, but not in starved bacteria incubated in PBS with the sufficient concentration of K^+^. Remarkably, the Kdp system was found upregulated in Mtb not only after relatively short (4 h) exposure to K^+^-free medium (4 h), (MacGilvary et al., 2019) but also after prolonged incubation under potassium deficiency for several weeks (Salina et al., 2014a; Ignatov et al., 2015). Another marked difference is that the dormancy regulator DosR was found activated both in transcriptome and proteome data only in long-stored Mab under potassium depletion, and not in starved Mab. Remarkably, dormant Mab exhibited a strong positive correlation between transcriptome and proteome changes indicating coordinated regulation at both levels, while starved bacteria showed a much weaker correlation between these two datasets (**Supplementary Figure 4**). We suggest that such discordance between differentially regulated genes and proteins in Mab after a 14-day incubation in PBS may reflect early dormant state in starved bacteria.

A comparison of the transcriptomic signatures of Mab subjected to various clinically relevant and stress conditions (**Table 1** and references therein) with those of dormant and starved Mab shows a significant overlap with bacteria survived in macrophages (Dubois et al., 2019), incubated in the artificial sputum medium (Miranda-CasoLuengo et al., 2016), under hypoxic conditions (Miranda-CasoLuengo et al., 2016; Simcox et al., 2023), and even upon exposure to transition metal cations (Liu et al., 2024) though to a lesser extent. The most common ‘metabolic features’ were the activation of lipid degradation and glyoxylate shunt with the repression of glycolytic reactions, cell division, and translation (**Table 1**).

Notably, dormant and starved Mab showed the down-regulation of proteins implicated in mycobacterial virulence and host-cell signaling modulation (MmpL and MmpS family), ‘host-pathogen’ interaction (YrbE family), and lipid transport (Mce family), both in transcriptome and proteome (**Figure 7**; **Supplementary Tables 4, 5, 6**). The suppression of proteins of Mce, MmpL, MmpS and YrbE families was proved to occur in intracellular Mab and in Mab grown the artificial sputum and (Miranda-CasoLuengo et al., 2016; Dubois et al., 2019). A question of whether suppressed *de novo* transcription, determined by ^3^H-uracil incorporation (**Figure 1B**), is sufficient to support an elevated amount of specific transcripts, particularly in dormant Mab, is a subject of considerable interest. Normally, individual transcripts in bacterial cells are known to have a very short lifetime (Anderson and Dunman, 2009; Bernstein et al., 2002; Hambraeus et al., 2003), however, the preservation of several individual mRNA molecules were demonstrated in rifampicin-treated *M. tuberculosis* cells (Hu et al., 2000). Remarkably, a significant increase in the average mRNA half-life in *M. tuberculosis* was shown under hypoxic conditions and at low cultivation temperature and (Rustad et al., 2013). Dormant Mtb cells were found to contain the low-abundant but stable transcriptome during persistence in the ‘zero-CFU’ state (Ignatov et al., 2015). Moreover, transcripts were found in endospores of *Bacillus* (Keijser et al., 2007; Segev et al., 2012); myxospores of myxobacteria (Muñoz-Dorado et al., 2019) differentiated small and large resting cells of *M. smegmatis* (Wu et al., 2016). The mechanism of preservation of transcripts in metabolically inactive cells by biocrystallization of nucleoid and temporary inactivation of RNAses due to their binding to small molecules, accompanied by dehydratation, was suggested recently (Suzina et al., 2019). Probably, these stored transcripts may contribute to the reversion of dormant Mab to growth under the appropriate conditions.

These data provide new insights into specific metabolic pathways that are crucial for long-term survival of Mab in NRS, when the efficacy of antimicrobial therapy is questionably. Further elucidation of the molecular mechanisms of maintenance of Mab viability during infection and adaptation to clinically relevant stress will suggest new targets for combating these persistent phenotypes and provide cues for the developing of new strategies to overcome mycobacterial infections.

## Data Availability

The original contributions presented in the study are included in the article/**Supplementary Material**. Further inquiries can be directed to the corresponding author.

## References

[B1] Aguilar-Ayala D. A. Palomino J. C. Vandamme P. Martin A. Gonzalez-y-Merchand J. A. (2017). Genetic regulation of Mycobacterium tuberculosis in a lipid-rich environment. Infect. Genet. Evol. 55, 392–402. doi: 10.1016/J.MEEGID.2016.10.015, PMID: 27771519

[B2] Amaral L. Martins M. Viveiros M. (2007). Enhanced killing of intracellular multidrug-resistant Mycobacterium tuberculosis by compounds that affect the activity of efflux pumps. J. Antimicrob. Chemother. 59, 1237–1246. doi: 10.1093/JAC/DKL500, PMID: 17218448

[B3] Anderson K. L. Dunman P. M. (2009). Messenger RNA Turnover Processes in Escherichia coli, Bacillus subtilis, and Emerging Studies in Staphylococcus aureus. Int. J. Microbiol. 2009, 525491. doi: 10.1155/2009/525491, PMID: 19936110 PMC2777011

[B4] Andrews S. (2010). FastQC: a quality control tool for high throughput sequence data. Available online at: http://www.bioinformatics.babraham.ac.uk/projects/fastqc (Accessed December 12, 2024).

[B5] Anuchin A. M. Mulyukin A. L. Suzina N. E. Duda V. I. El-Registan G. I. Kaprelyants A. S. (2009). Dormant forms of Mycobacterium smegmatis with distinct morphology. Microbiology 155, 1071–1079. doi: 10.1099/MIC.0.023028-0, PMID: 19332809

[B6] Bacon J. James B. W. Wernisch L. Williams A. Morley K. A. Hatch G. J. . (2004). The influence of reduced oxygen availability on pathogenicity and gene expression in Mycobacterium tuberculosis. Tuberculosis 84, 205–217. doi: 10.1016/j.tube.2003.12.011, PMID: 15207490

[B7] Bernstein J. A. Khodursky A. B. Lin P. H. Lin-Chao S. Cohen S. N. (2002). Global analysis of mRNA decay and abundance in Escherichia coli at single-gene resolution using two-color fluorescent DNA microarrays. Proc. Natl. Acad. Sci. U.S.A. 99, 9697–9702. doi: 10.1073/pnas.112318199, PMID: 12119387 PMC124983

[B8] Berube B. J. Castro L. Russell D. Ovechkina Y. Parish T. (2018). Novel screen to assess bactericidal activity of compounds against non-replicating mycobacterium abscessus. Front. Microbiol. 9. doi: 10.3389/FMICB.2018.02417, PMID: 30364170 PMC6191478

[B9] Betts J. C. Lukey P. T. Robb L. C. McAdam R. A. Duncan K. (2002). Evaluation of a nutrient starvation model of Mycobacterium tuberculosis persistence by gene and protein expression profiling. Mol. Microbiol. 43, 717–731. doi: 10.1046/j.1365-2958.2002.02779.x, PMID: 11929527

[B10] Chao M. C. Rubin E. J. (2010). Letting sleeping dos lie: does dormancy play a role in tuberculosis? Annu. Rev. Microbiol. 64, 293–311. doi: 10.1146/ANNUREV.MICRO.112408.134043, PMID: 20825351

[B11] Chen Y. Hagopian B. Tan S. (2025). Cholesterol metabolism and intrabacterial potassium homeostasis are intrinsically related in Mycobacterium tuberculosis. PloS Pathog. 21(5), e1013207. doi: 10.1371/JOURNAL.PPAT.1013207, PMID: 40402977 PMC12136442

[B12] Cocorullo M. Stamilla A. Recchia D. Marturano M. C. Maci L. Stelitano G. (2025). Mycobacterium abscessus virulence factors: an overview of un-explored therapeutic options. Int. J. Mol. Sci. 26(7), 3247. doi: 10.3390/IJMS26073247, PMID: 40244091 PMC11990050

[B13] de Man J. C. (1975). The probability of most probable numbers. Eur. J. Appl. Microbiol. 1, 67–78. doi: 10.1007/BF01880621

[B14] Dubois V. Pawlik A. Bories A. Le Moigne V. Sismeiro O. Legendre R. . (2019). Mycobacterium abscessus virulence traits unraveled by transcriptomic profiling in amoeba and macrophages. PloS Pathog. 15(11), e1008069. doi: 10.1371/JOURNAL.PPAT.1008069, PMID: 31703112 PMC6839843

[B15] Epstein W. (2003). The roles and regulation of potassium in bacteria. Prog. Nucleic Acid Res. Mol. Biol. 75, 293–320. doi: 10.1016/S0079-6603(03)75008-9, PMID: 14604015

[B16] Feilcke R. Eckenstaler R. Lang M. Richter A. Imming P. (2025). A simple *in vitro* method to determine bactericidal activity against mycobacterium abscessus under hypoxic conditions. Antibiot. (Basel Switzerland) 14 (3), 299. doi: 10.3390/ANTIBIOTICS14030299, PMID: 40149109 PMC11939544

[B17] Gries C. M. Bose J. L. Nuxoll A. S. Fey P. D. Bayles K. W. (2013). The Ktr potassium transport system in Staphylococcus aureus and its role in cell physiology, antimicrobial resistance and pathogenesis. Mol. Microbiol. 89, 760–773. doi: 10.1111/MMI.12312, PMID: 23815639 PMC3754831

[B18] Hambraeus G. von Wachenfeldt C. Hederstedt L. (2003). Genome-wide survey of mRNA half-lives in Bacillus subtilis identifies extremely stable mRNAs. Mol. Genet. Genomics 269, 706–714. doi: 10.1007/s00438-003-0883-6, PMID: 12884008

[B19] Hampshire T. Soneji S. Bacon J. James B. W. Hinds J. Laing K. . (2004). Stationary phase gene expression of Mycobacterium tuberculosis following a progressive nutrient depletion: A model for persistent organisms? Tuberculosis 84, 228–238. doi: 10.1016/j.tube.2003.12.010, PMID: 15207492 PMC3195342

[B20] Hu Y. Mangan J. A. Dhillon J. Sole K. M. Mitchison D. A. Butcher P. D. . (2000). Detection of mRNA transcripts and active transcription in persistent Mycobacterium tuberculosis induced by exposure to rifampin or pyrazinamide. J. Bacteriol. 182, 6358–6365. doi: 10.1128/JB.182.22.6358-6365.2000, PMID: 11053379 PMC94781

[B21] Huerta-Cepas J. Szklarczyk D. Forslund K. Cook H. Heller D. Walter M. C. . (2016). eggNOG 4.5: a hierarchical orthology framework with improved functional annotations for eukaryotic, prokaryotic and viral sequences. Nucleic Acids Res. 44, D286–D293. doi: 10.1093/NAR/GKV1248, PMID: 26582926 PMC4702882

[B22] Ignatov D. V. Salina E. G. Fursov M. V. Skvortsov T. A. Azhikina T. L. Kaprelyants A. S. (2015). Dormant non-culturable Mycobacterium tuberculosis retains stable low-abundant mRNA. BMC Genomics 16, 1–13. doi: 10.1186/s12864-015-2197-6, PMID: 26573524 PMC4647672

[B23] Johansen M. D. Herrmann J. L. Kremer L. (2020). Non-tuberculous mycobacteria and the rise of Mycobacterium abscessus. Nat. Rev. Microbiol. 18, 392–407. doi: 10.1038/S41579-020-0331-1, PMID: 32086501

[B24] Keijser B. J. Ter Beek A. Rauwerda H. Schuren F. Montijn R. van der Spek H. . (2007). Analysis of temporal gene expression during Bacillus subtilis spore germination and outgrowth. J. Bacteriol. 189, 3624–3634. doi: 10.1128/JB.01736-06, PMID: 17322312 PMC1855883

[B25] Kovalchuk S. I. Jensen O. N. Rogowska-Wrzesinska A. (2019). FlashPack: fast and simple preparation of ultrahigh-performance capillary columns for LC-MS. Mol. Cell. Proteomics 18, 383–390. doi: 10.1074/MCP.TIR118.000953, PMID: 30373789 PMC6356079

[B26] Kundu M. Basu J. (2021). Applications of transcriptomics and proteomics for understanding dormancy and resuscitation in mycobacterium tuberculosis. Front. Microbiol. 12. doi: 10.3389/FMICB.2021.642487, PMID: 33868200 PMC8044303

[B27] Kurihara Y. Shimizu A. Ozuru R. Yoshimura M. Chou B. Itoh R. . (2025). Mycobacterium abscessus resides within lipid droplets and acquires a dormancy-like phenotype in adipocytes. Biochem. Biophys. Res. Commun. 758, 151645. doi: 10.1016/J.BBRC.2025.151645, PMID: 40120350

[B28] Langmead B. Salzberg S. L. (2012). Fast gapped-read alignment with Bowtie 2. Nat. Methods 9, 357–359. doi: 10.1038/nmeth.1923, PMID: 22388286 PMC3322381

[B29] Lewis K. (2007). Persister cells, dormancy and infectious disease. Nat. Rev. Microbiol. 5, 48–56. doi: 10.1038/NRMICRO1557, PMID: 17143318

[B30] Liao Y. Smyth G. K. Shi W. (2014). FeatureCounts: An efficient general purpose program for assigning sequence reads to genomic features. Bioinformatics 30, 923–930. doi: 10.1093/bioinformatics/btt656, PMID: 24227677

[B31] Liu Y. Ho K. K. Su J. Gong H. Chang A. C. Lu S. (2013). Potassium transport of Salmonella is important for type III secretion and pathogenesis. Microbiology 159, 1705–1719. doi: 10.1099/MIC.0.068700-0, PMID: 23728623 PMC4089031

[B32] Liu Y. Murphy K. Fernandes N. Moore R. E. T. Pennisi I. Williams R. . (2024). Transition metal homoeostasis is key to metabolism and drug tolerance of Mycobacterium abscessus. NPJ Antimicrob. Resist. 2(1), 25. doi: 10.1038/S44259-024-00042-7, PMID: 39359892 PMC11442307

[B33] Love M. I. Huber W. Anders S. (2014). Moderated estimation of fold change and dispersion for RNA-seq data with DESeq2. Genome Biol. 15, 550. doi: 10.1186/s13059-014-0550-8, PMID: 25516281 PMC4302049

[B34] Ma B. Zhang K. Hendrie C. Liang C. Li M. Doherty-Kirby A. . (2003). PEAKS: powerful software for peptide *de novo* sequencing by tandem mass spectrometry. Rapid Commun. Mass Spectrom. 17, 2337–2342. doi: 10.1002/RCM.1196, PMID: 14558135

[B35] MacGilvary N. J. Kevorkian Y. L. Tan S. (2019). Potassium response and homeostasis in Mycobacterium tuberculosis modulates environmental adaptation and is important for host colonization. PloS Pathog. 15(2), e1007591. doi: 10.1371/JOURNAL.PPAT.1007591, PMID: 30716121 PMC6375644

[B36] Medjahed H. Gaillard J. L. Reyrat J. M. (2010). Mycobacterium abscessus: a new player in the mycobacterial field. Trends Microbiol. 18, 117–123. doi: 10.1016/J.TIM.2009.12.007, PMID: 20060723

[B37] Miranda-CasoLuengo A. A. Staunton P. M. Dinan A. M. Lohan A. J. Loftus B. J. (2016). Functional characterization of the Mycobacterium abscessus genome coupled with condition specific transcriptomics reveals conserved molecular strategies for host adaptation and persistence. BMC Genomics 17, 553. doi: 10.1186/S12864-016-2868-Y, PMID: 27495169 PMC4974804

[B38] Mootha V. K. Lindgren C. M. Eriksson K. F. Subramanian A. Sihag S. Lehar J. . (2003). PGC-1alpha-responsive genes involved in oxidative phosphorylation are coordinately downregulated in human diabetes. Nat. Genet. 34, 267–273. doi: 10.1038/NG1180, PMID: 12808457

[B39] Mulyukin A. L. Kudykina Y. K. Shleeva M. O. Anuchin A. M. Suzina N. E. Danilevich V. N. . (2010). Intraspecies diversity of dormant forms of Mycobacterium smegmatis. Microbiology 79, 461–471. doi: 10.1134/S0026261710040089/METRICS

[B40] Mulyukin A. L. Recchia D. Kostrikina N. A. Artyukhina M. V. Martini B. A. Stamilla A. . (2023). Distinct effects of moxifloxacin and bedaquiline on growing and “Non-culturable” mycobacterium abscessus. Microorganisms 11(11), 2690. doi: 10.3390/MICROORGANISMS11112690, PMID: 38004702 PMC10673116

[B41] Muñoz-Dorado J. Moraleda-Muñoz A. Marcos-Torres F. J. Contreras-Moreno F. J. Martin-Cuadrado A. B. Schrader J. M. . (2019). Transcriptome dynamics of the Myxococcus xanthus multicellular developmental program. Elife 8, e50374. doi: 10.7554/ELIFE.50374, PMID: 31609203 PMC6791715

[B42] Perez-Riverol Y. Csordas A. Bai J. Bernal-Llinares M. Hewapathirana S. Kundu D. J. . (2019). The PRIDE database and related tools and resources in 2019: improving support for quantification data. Nucleic Acids Res. 47, D442–D450. doi: 10.1093/NAR/GKY1106, PMID: 30395289 PMC6323896

[B43] Roux A. L. Viljoen A. Bah A. Simeone R. Bernut A. Laencina L. . (2016). The distinct fate of smooth and rough Mycobacterium abscessus variants inside macrophages. Open Biol. 6(11)160185. doi: 10.1098/RSOB.160185, PMID: 27906132 PMC5133439

[B44] Rustad T. R. Minch K. J. Brabant W. Winkler J. K. Reiss D. J. Baliga N. S. . (2013). Global analysis of mRNA stability in Mycobacterium tuberculosis. Nucleic Acids Res. 41, 509–517. doi: 10.1093/nar/gks1019, PMID: 23125364 PMC3592478

[B45] Rustad T. R. Roberts D. M. Liao R. P. Sherman D. R. (2008). Isolation of mycobacterial RNA. Methods Mol. Biol. 465, 13–22. doi: 10.1007/978-1-59745-207-6_2, PMID: 20560069

[B46] Salina E. G. Martini B. A. Sorokin V. V. Mulyukin A. L. (2024). Fate of *in vitro* cultured Mycobacterium abscessus populations when exposed to moxifloxacin. Front. Microbiol. 15. doi: 10.3389/FMICB.2024.1494147, PMID: 39669783 PMC11635960

[B47] Salina E. Ryabova O. Kaprelyants A. Makarov V. (2014b). New 2-thiopyridines as potential candidates for killing both actively growing and dormant Mycobacterium tuberculosis cells. Antimicrob. Agents Chemother. 58, 55–60. doi: 10.1128/AAC.01308-13, PMID: 24126578 PMC3910762

[B48] Salina E. G. Waddell S. J. Hoffmann N. Rosenkrands I. Butcher P. D. Kaprelyants A. S. (2014a). Potassium availability triggers Mycobacterium tuberculosis transition to, and resuscitation from, non-culturable (dormant) states. Open Biol. 4, 140106. doi: 10.1098/rsob.140106, PMID: 25320096 PMC4221891

[B49] Segev E. Smith Y. Ben-Yehuda S. (2012). RNA dynamics in aging bacterial spores. Cell 148, 139–149. doi: 10.1016/J.CELL.2011.11.059, PMID: 22209493

[B50] Shleeva M. O. Kudykina Y. K. Vostroknutova G. N. Suzina N. E. Mulyukin A. L. Kaprelyants A. S. (2011). Dormant ovoid cells of Mycobacterium tuberculosis are formed in response to gradual external acidification. Tuberculosis 91, 146–154. doi: 10.1016/j.tube.2010.12.006, PMID: 21262587

[B51] Shleeva M. O. Trutneva K. A. Demina G. R. Zinin A. I. Sorokoumova G. M. Laptinskaya P. K. . (2017). Free trehalose accumulation in dormant mycobacterium smegmatis cells and its breakdown in early resuscitation phase. Front. Microbiol. 8. doi: 10.3389/FMICB.2017.00524, PMID: 28424668 PMC5371599

[B52] Simcox B. S. Tomlinson B. R. Shaw L. N. Rohde K. H. (2023). Mycobacterium abscessus DosRS two-component system controls a species-specific regulon required for adaptation to hypoxia. Front. Cell. Infect. Microbiol. 13. doi: 10.3389/FCIMB.2023.1144210, PMID: 36968107 PMC10034137

[B53] Stingl K. Brandt S. Uhlemann E. M. Schmid R. Altendorf K. Zeilinger C. . (2007). Channel-mediated potassium uptake in Helicobacter pylori is essential for gastric colonization. EMBO J. 26, 232–241. doi: 10.1038/SJ.EMBOJ.7601471, PMID: 17159901 PMC1782367

[B54] Subramanian A. Tamayo P. Mootha V. K. Mukherjee S. Ebert B. L. Gillette M. A. . (2005). Gene set enrichment analysis: a knowledge-based approach for interpreting genome-wide expression profiles. Proc. Natl. Acad. Sci. U. S. A. 102, 15545–15550. doi: 10.1073/PNAS.0506580102, PMID: 16199517 PMC1239896

[B55] Suzina N. E. Polivtseva V. N. Shorokhova A. P. (2019). Ultrastructural organization and enzymes of the antioxidant defense system in the dormant cells of gram-negative bacteria *Stenotrophomonas* sp. strain FM3 and *Morganella morganii* subsp. *sibonii* strain FF1. Microbiology 88, 183–190. doi: 10.1134/S0026261719020115

[B56] Tan S. (2021). Abundant monovalent ions as environmental signposts for pathogens during host colonization. Infect. Immun. 89(4), e00641-20. doi: 10.1128/IAI.00641-20, PMID: 33526568 PMC8090958

[B57] Taneja N. K. Dhingra S. Mittal A. Naresh M. Tyagi J. S. (2010). Mycobacterium tuberculosis transcriptional adaptation, growth arrest and dormancy phenotype development is triggered by vitamin C. PloS One 5(5), e10860. doi: 10.1371/JOURNAL.PONE.0010860, PMID: 20523728 PMC2877710

[B58] Touré H. Galindo L. A. Lagune M. Glatigny S. Waterhouse R. M. Guénal I. . (2023). Mycobacterium abscessus resists the innate cellular response by surviving cell lysis of infected phagocytes. PloS Pathog. 19(3), e1011257. doi: 10.1371/JOURNAL.PPAT.1011257, PMID: 36972320 PMC10079227

[B59] Trutneva K. Shleeva M. Nikitushkin V. Demina G. Kaprelyants A. (2018). Protein composition of Mycobacterium smegmatis differs significantly between active cells and dormant cells with ovoid morphology. Front. Microbiol. 9. doi: 10.3389/FMICB.2018.02083/FULL, PMID: 30233550 PMC6131537

[B60] Vang C. K. Dawrs S. N. Oberlag N. M. Gilmore A. E. Hasan N. A. Honda J. R. (2022). Comparative survival of environmental and clinical Mycobacterium abscessus isolates in a variety of diverse host cells. J. Appl. Microbiol. 132, 3302–3314. doi: 10.1111/JAM.15416, PMID: 34919308 PMC9306708

[B61] Verma A. Ghoshal A. Dwivedi V. P. Bhaskar A. (2022). Tuberculosis: The success tale of less explored dormant Mycobacterium tuberculosis. Front. Cell. Infect. Microbiol. 12. doi: 10.3389/FCIMB.2022.1079569/XML/NLM, PMID: 36619761 PMC9813417

[B62] Viljoen A. Blaise M. de Chastellier C. Kremer L. (2016). MAB_3551c encodes the primary triacylglycerol synthase involved in lipid accumulation in Mycobacterium abscessus. Mol. Microbiol. 102, 611–627. doi: 10.1111/MMI.13482, PMID: 27513974

[B63] Voskuil M. I. Visconti K. C. Schoolnik G. K. (2004). Mycobacterium tuberculosis gene expression during adaptation to stationary phase and low-oxygen dormancy. Tuberculosis 84, 218–227. doi: 10.1016/j.tube.2004.02.003, PMID: 15207491

[B64] Wiese M. Seydel U. (1996). Drug effects on intracellular mycobacteria determined by mass spectrometric analysis of the Na(+)-to-K+ ratios of individual bacterial organisms. Antimicrob. Agents Chemother. 40, 2047–2053. doi: 10.1128/AAC.40.9.2047, PMID: 8878579 PMC163471

[B65] Wood J. M. (2011). Bacterial osmoregulation: a paradigm for the study of cellular homeostasis. Annu. Rev. Microbiol. 65, 215–238. doi: 10.1146/ANNUREV-MICRO-090110-102815, PMID: 21663439

[B66] Wu M. L. Gengenbacher M. Chung J. C. Chen S. L. Mollenkopf H. J. Kaufmann S. H. . (2016). Developmental transcriptome of resting cell formation in Mycobacterium smegmatis. BMC Genomics 17, 837. doi: 10.1186/s12864-016-3190-4, PMID: 27784279 PMC5081680

[B67] Yam Y. K. Alvarez N. Go M. L. Dick T. (2020). Extreme drug tolerance of mycobacterium abscessus “Persisters. Front. Microbiol. 11. doi: 10.3389/FMICB.2020.00359, PMID: 32194537 PMC7064438

[B68] Zhang Y. (2004). Persistent and dormant tubercle bacilli and latent tuberculosis. Front. Biosci. 9, 1136–1156. doi: 10.2741/1291, PMID: 14977534

