# Supplementary material for: Insights into Mycobacterium abscessus survival under prolonged potassium deficiency and starvation
Source: Front Cell Infect Microbiol. 2025 Nov 26;15:1668407. doi: 10.3389/fcimb.2025.1668407 (PMC12689514; doi:10.3389/fcimb.2025.1668407)
Supplement: Supplementary file 1 [file DataSheet1.pdf]

Supplementary Material

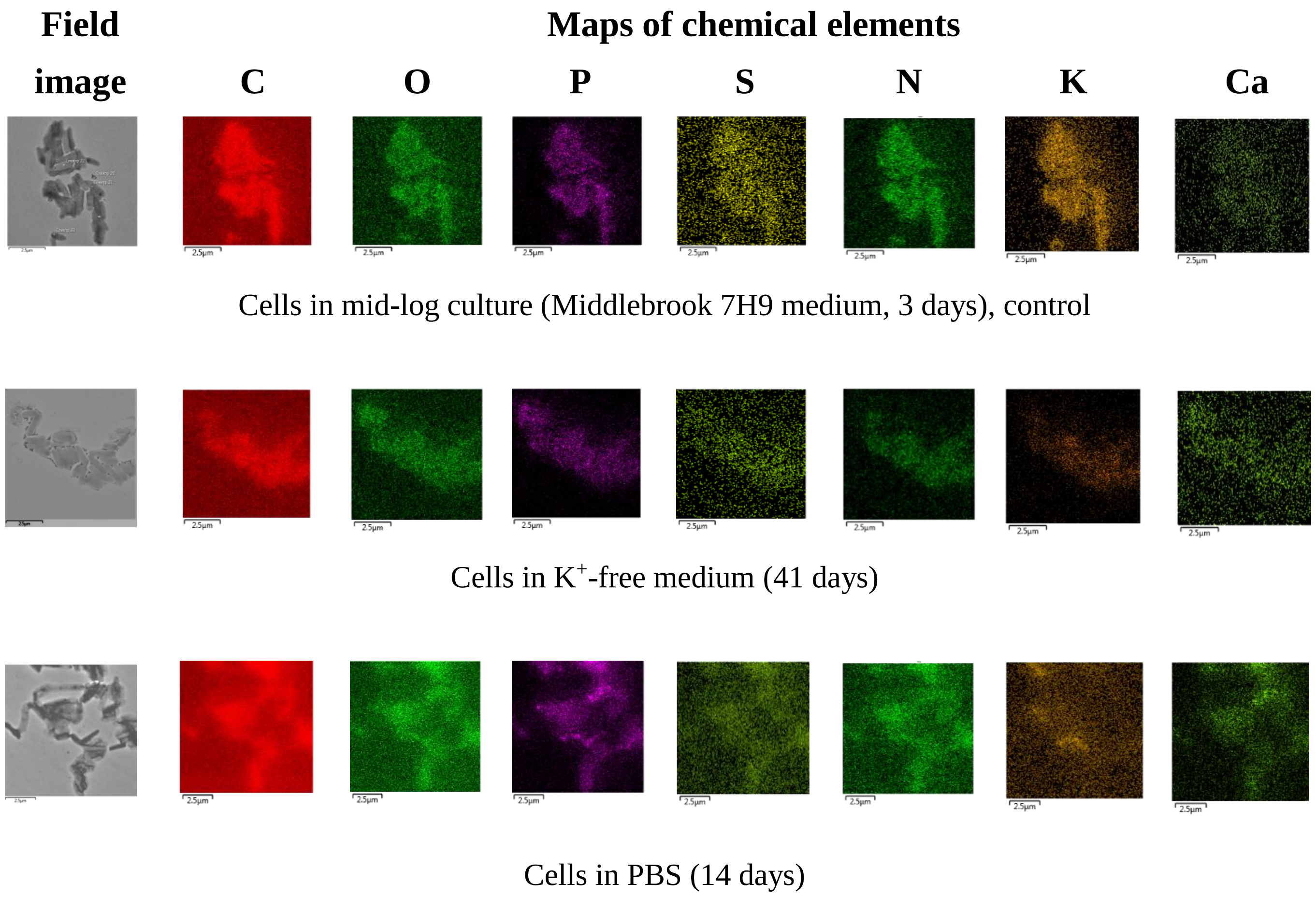

Supplementary Figure 1. TEM-EDX analysis: images of fields with Mab cells and colored maps of chemical elements. Bars, 2.5 µm

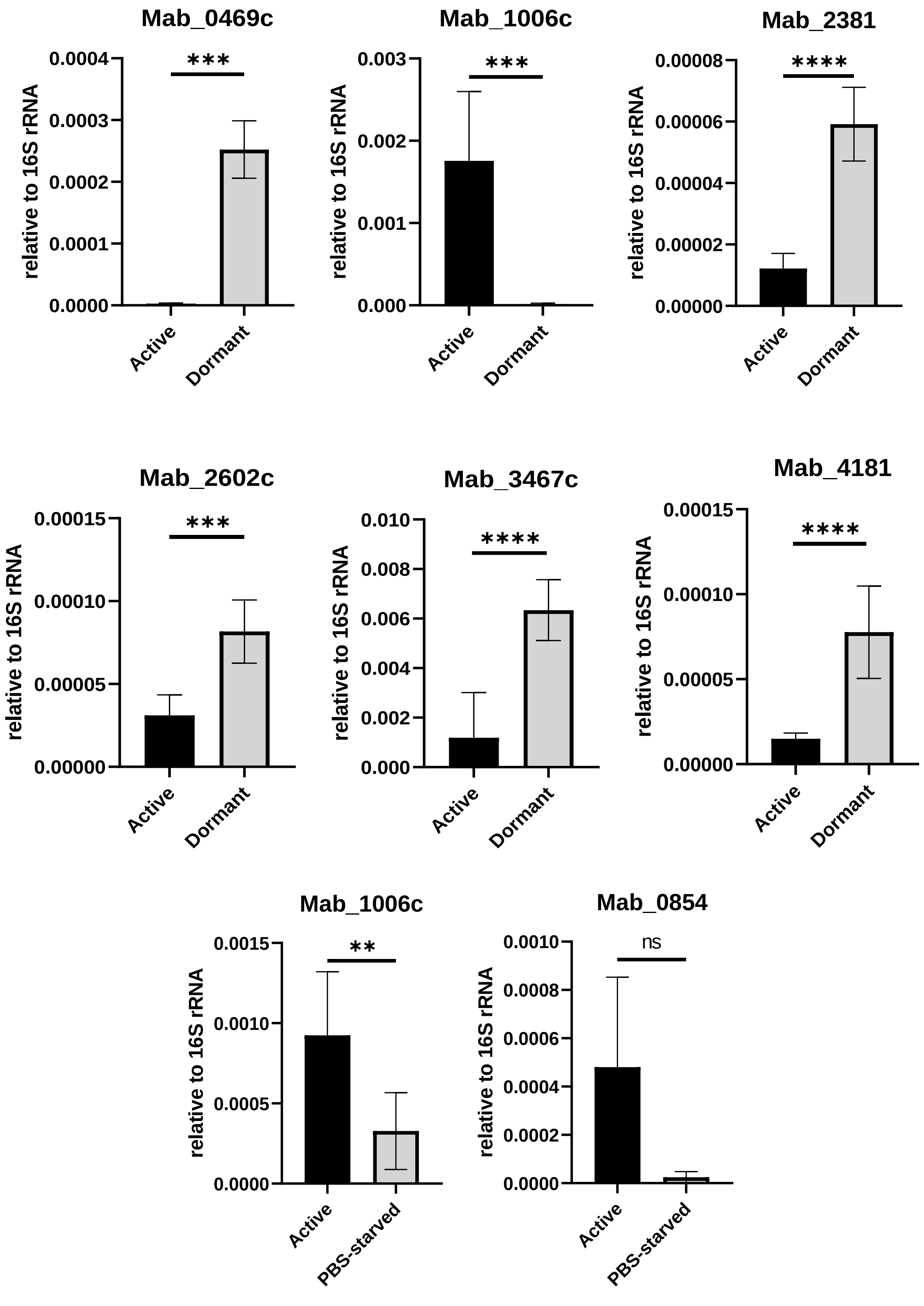

Supplementary Figure 2. Confirmation of expression of several DEG by qPCR. The data are normalized to 16S rRNA transcription level. **p<0.01, ***p<0.001, ****p<0.0001.

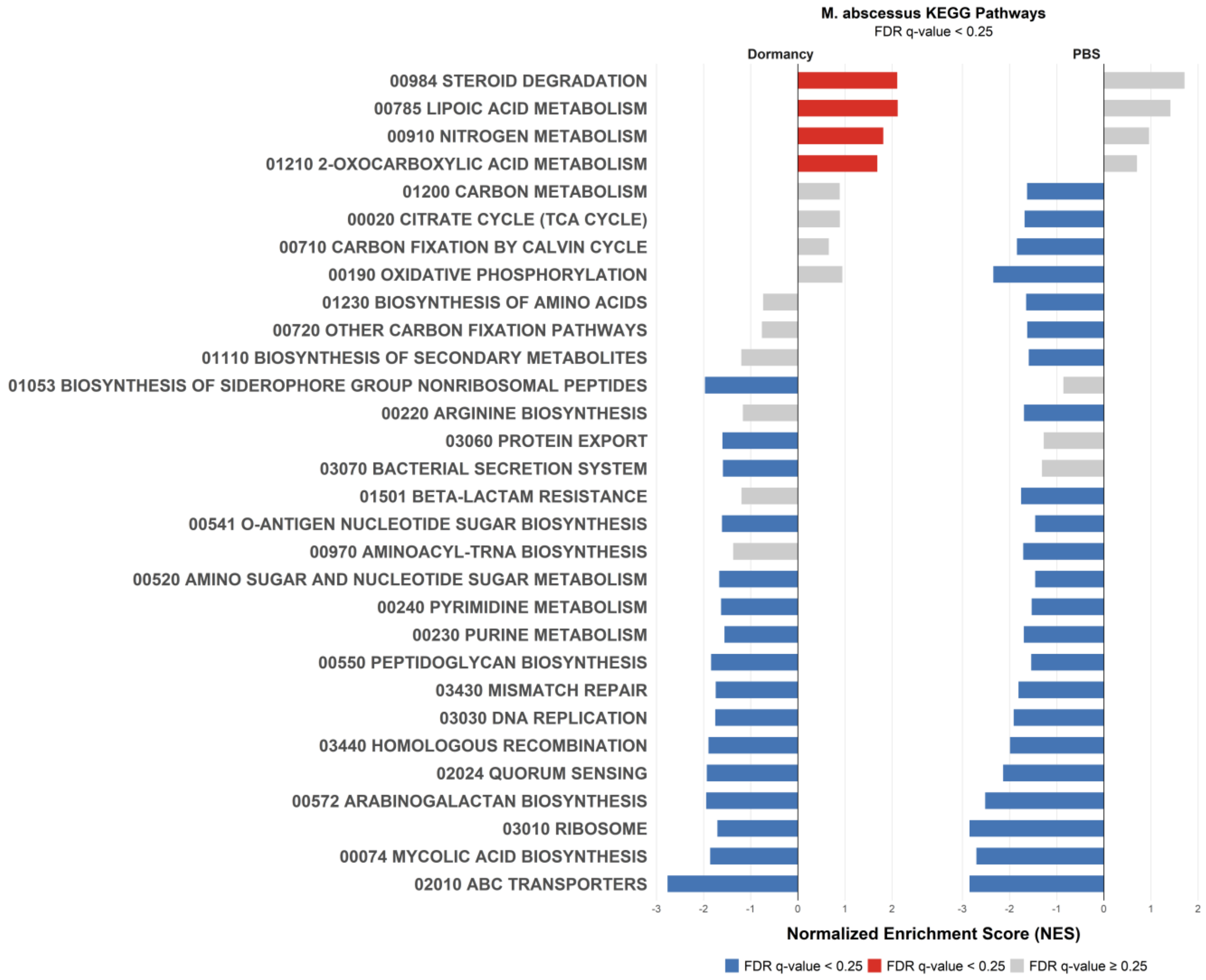

Supplementary Figure 3. Gene set enrichment analysis (GSEA) results comparing differentially expressed KEGG pathways in dormant and starved versus mid-log active Mab. Pathways with significant differences (FDR q-value < 0.25) are shown with red bars indicating enrichment under potassium deficiency and nutrient starvation and blue bars indicating down-regulation in these conditions. Gray bars represent non-significant pathways. Dormancy is characterized by significant up-regulation of steroid degradation, lipoic acid metabolism, nitrogen metabolism, and 2-oxocarboxylic acid metabolism pathways. Conversely, both dormancy and starvation conditions show pronounced down-regulation of key cellular processes including ABC transporters, mycolic acid biosynthesis, ribosomal activity, arabinogalactan biosynthesis, DNA replication, peptidoglycan biosynthesis, and nucleotide metabolism.

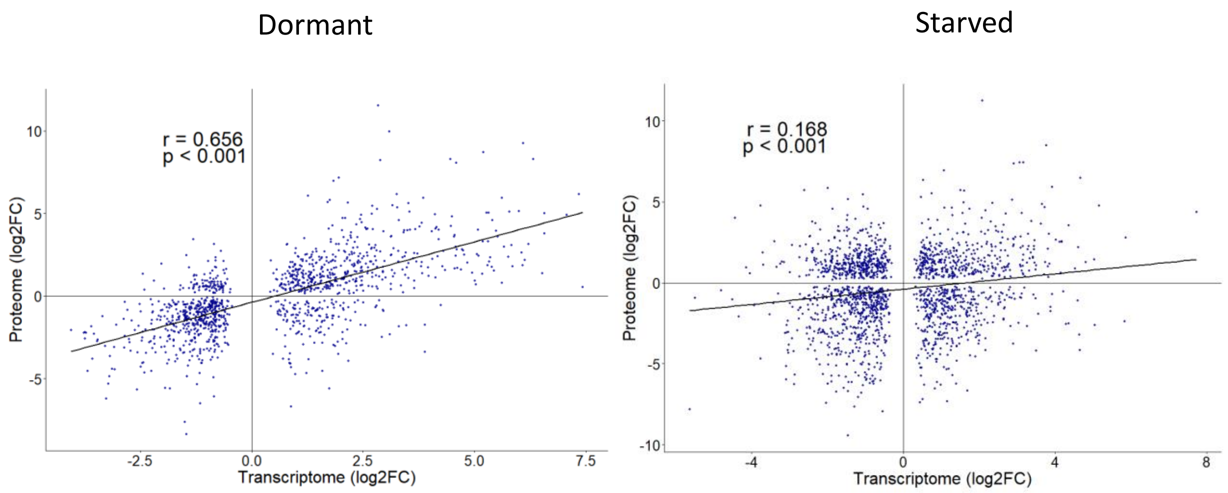

Supplementary Figure 4. Correlation between transcriptome and proteome changes in dormant and starved Mab. Scatter plots showing the correlation between RNA-seq log2 fold changes (log2FC) and proteome log2 fold changes for genes with statistically significant changes in both datasets. Each point represents an individual gene. (Left) 41-day dormant Mab (Pearson's $r = 0.656$, $p < 0.001$). (Right) 14-day PBS-starved Mab (Pearson's $r = 0.168$, $p < 0.001$). Black lines represent linear regression fits.

Supplementary Table 1. Oligonucleotides used in the study.

**Name**	**Sequence**
Mab_3467c_qf	CAACAGGTGTTGGGTACTGCG
Mab_3467c_qr	ACCGTGACCACGTTGTGTTCG
Mab_2602c_qf	GCAGGTGAACTCTCGGTCAATG
Mab_2602c_qr	ACCAGCTGTTCGACATGTTCG
Mab_1006c_qf	CCTTCGGCGACCTCATCAAAG
Mab_1006c_qr	TCTTGGTGTCGAGCTGGTCAG
Mab_0854_qf	ACATCAAACACGCCGGGTTGC
Mab_0854_qr	TGAATTGCTGGTGCCACCACG
Mab_0469c_qf	GAAGATAGCGATGTTGGCGGC
Mab_0469c_qr	GTTCGCATAGCAAACCGGAGG
Mab_2381_qf	AGGATCAAAGGACCGCGCAA
Mab_2381_qr	GGAGTTCCTCAAGGAATGCC
Mab_4181_qf	GCGGAGGCGGTCTACATATT
Mab_4181_qr	AGCATGACGACGGAATCCTTG

Supplementary Table 2. Estimated parameters of K^+ abundance and distribution between cells and surrounding medium.

Field	Map for potassium	Proportions of cells with normal (N), depleted (D), and subzero (sZ) K^+ levels*	Range of K^+ abundance index, I_{K+} 10^{-3}×C/µm^2×N_{cells}	Range of intracellular to extracellular K^+ ratio, K_{int}/K_{extr}
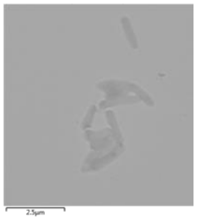	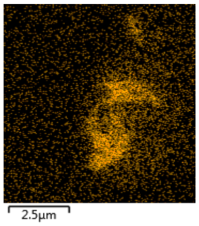	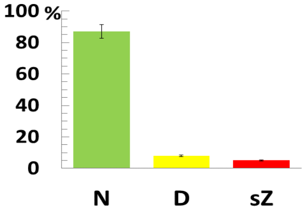	0.15 – 0.29 (median 0.22)	1 – 15 (median 5.9)
Cells in mid-log culture (Middlebrook 7H9 medium, 3 days), control				
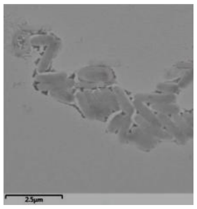	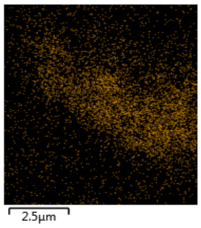	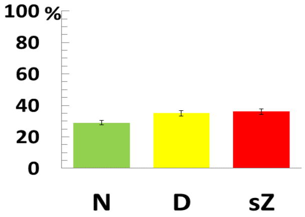	0.002 – 0.14 (median 0.01)	6 – 1000 (median 130)
Cells in K^+-free medium (44 days)				
(*for all images and maps based on differential counting for 168 – 216 cells)				
**Heterogeneity of cell subpopulations (clumps) under potassium deficiency**				
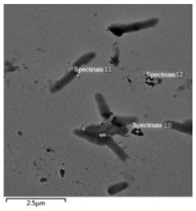 subpopulation 1	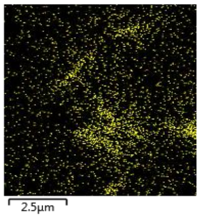	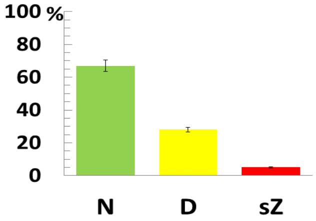	0.14	6
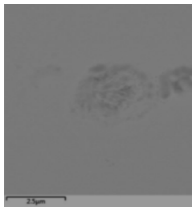 subpopulation 2	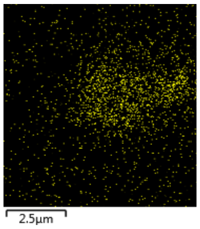	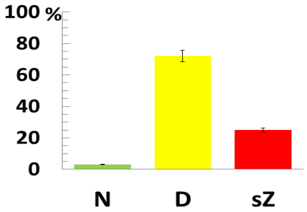	0.09	130
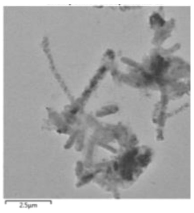 subpopulation 3	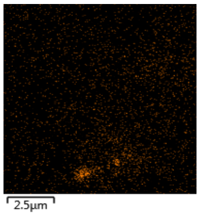	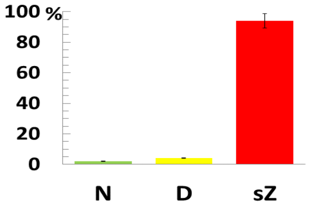	0.002	>10^3

Supplementary Table 3. Mapping statistics for each of the sequenced samples. The percentage of reads mapped to coding sequences (CDS), rRNA and tRNA are shown. Data provided in MS Excel format.

Supplementary Table 4. Differential expression of protein-coding genes in dormant and starved Mab in comparison to active bacteria. The analysis of differential expression was performed using the DESeq2 software package according to the following criteria: adjusted *p*-value < 0.01 and $|\log_2$ fold change ($\log_2$ FC)$|$ value ≥ 2.0. Each sample was analyzed in three biological replicates. Data provided in MS Excel format.

Supplementary Table 5. Differential expression of proteins in dormant and starved Mab in comparison to active bacteria. MS raw files were analyzed using PEAKS Studio 8.5 (Bioinformatics Solutions Inc., Canada) and peak lists were searched against UniProtKB/TrEMBLE FASTA (canonical and isoform; version of November 2023) for *M. abscessus* ATCC 19977 with methionine oxidation and asparagine and glutamine deamidation as variable modifications. False discovery rate was set to 0.01 and $|\log_2$ fold change ($\log_2$ FC)$|$ value ≥ 2.0. Each sample was analyzed in three biological replicates. Data provided in MS Excel format.

Supplementary Table 6. Comparison of differentially expressed genes and proteins for dormant and starved Mab. Data provided in MS Excel format.
